# Characteristics, Progression, and Output of Randomized Platform Trials: A Systematic Review

**DOI:** 10.1001/jamanetworkopen.2024.3109

**Published:** 2024-03-20

**Authors:** Alexandra Griessbach, Christof Manuel Schönenberger, Ala Taji Heravi, Viktoria Gloy, Arnav Agarwal, Tim Jonas Hallenberger, Stefan Schandelmaier, Perrine Janiaud, Alain Amstutz, Manuela Covino, David Mall, Benjamin Speich, Matthias Briel

**Affiliations:** 1 CLEAR Methods Center, Division of Clinical Epidemiology, Department of Clinical Research, University Hospital Basel, University of Basel, Basel, Switzerland; 2 Department of Medicine, McMaster University, Hamilton, Ontario, Canada; 3 Department of Health Research Methods, Evidence and Impact, McMaster University, Hamilton, Ontario, Canada; 4 Department of Neurosurgery, University Hospital Basel, Basel, Switzerland; 5 Pragmatic Evidence Lab, Research Center for Clinical Neuroimmunology and Neuroscience Basel (RC2NB), University Hospital Basel and University of Basel, Basel, Switzerland

## Abstract

**Importance:**

Platform trials have become increasingly common, and evidence is needed to determine how this trial design is actually applied in current research practice.

**Objective:**

To determine the characteristics, progression, and output of randomized platform trials.

**Evidence Review:**

In this systematic review of randomized platform trials, Medline, Embase, Scopus, trial registries, gray literature, and preprint servers were searched, and citation tracking was performed in July 2022. Investigators were contacted in February 2023 to confirm data accuracy and to provide updated information on the status of platform trial arms. Randomized platform trials were eligible if they explicitly planned to add or drop arms. Data were extracted in duplicate from protocols, publications, websites, and registry entries. For each platform trial, design features such as the use of a common control arm, use of nonconcurrent control data, statistical framework, adjustment for multiplicity, and use of additional adaptive design features were collected. Progression and output of each platform trial were determined by the recruitment status of individual arms, the number of arms added or dropped, and the availability of results for each intervention arm.

**Findings:**

The search identified 127 randomized platform trials with a total of 823 arms; most trials were conducted in the field of oncology (57 [44.9%]) and COVID-19 (45 [35.4%]). After a more than twofold increase in the initiation of new platform trials at the beginning of the COVID-19 pandemic, the number of platform trials has since declined. Platform trial features were often not reported (not reported: nonconcurrent control, 61 of 127 [48.0%]; multiplicity adjustment for arms, 98 of 127 [77.2%]; statistical framework, 37 of 127 [29.1%]). Adaptive design features were only used by half the studies (63 of 127 [49.6%]). Results were available for 65.2% of closed arms (230 of 353). Premature closure of platform trial arms due to recruitment problems was infrequent (5 of 353 [1.4%]).

**Conclusions and Relevance:**

This systematic review found that platform trials were initiated most frequently during the COVID-19 pandemic and declined thereafter. The reporting of platform features and the availability of results were insufficient. Premature arm closure for poor recruitment was rare.

## Key Points

**Question:**

What are the characteristics, progression, and output of randomized platform trials?

**Findings:**

In this systematic review of 127 platform trials with a total of 823 arms, primarily in the fields of oncology and COVID-19, the adpative features of the trials were often poorly reported and only used in 49.6% of all trials; results were available for only 65.2% of completed trial arms.

**Meaning:**

The planning and reporting of platform features and the availability of results were insufficient in randomized platform trials.

## Teaser

This systematic review assesses the characteristics, progression, and output of randomized platform trials in current research practice.

## Introduction

Randomized clinical trials (RCTs) are the criterion standard for evaluating health care interventions. However, RCTs are criticized for being slow, inflexible, inefficient, and costly.^1,2,3,4,5,6^ The platform trial design^7^ may overcome some of the challenges associated with traditional RCTs.^5,8^

In the literature, the definition of platform trials is inconsistent.^7,9,10,11,12,13,14,15,16^ Common characteristics of platform trials include the simultaneous assessment of multiple interventions, as well as the ability to drop ineffective interventions or add promising new interventions (arms).^10,13,17,18,19,20^ Platform trial planning and conduct require consideration of their unique design features, methodological framework, and level of sophistication. This planning includes the potential use of a common control arm, nonconcurrent control data, the statistical framework (bayesian and/or frequentist), in silico trials (simulations), and the use of additional adaptive design features, such as response adaptive randomization (RAR; the change of the randomization ratio based on data collected during the trial), sample size reassessment, seamless design (seamless study phase transition), and adaptive enrichment (modification of eligibility criteria).^9,11,16^ Platform trials are stipulated to be more time efficient and cost efficient and are able to increase the output of the trial, benefiting both patients and researchers.^8,9,17^ Further potential benefits include the use of regulatory documentation (master protocol) and contracts beyond 1 trial and its respective duration,^8^ quick initiation of new sites and intervention arms,^21^ reuse of established infrastructure,^22^ and quick study phase transition.^22^

Empirical evidence about platform trials is needed to gain insight into the actual application of this design in clinical research practice and to learn about its benefits and pitfalls, so that the planning and conduct of platform trials can be further improved. Previous systematic reviews on platform trials are outdated^13,14^; are restricted to the late-phase, multiarm, multistage design or COVID-19 trials^23,24^; only investigated a small number of distinct platform trial features^23^; or did not consider the output of platform trials in terms of completed, prematurely closed, and published trial arms.^25^ A comprehensive overview is currently lacking. We specifically wondered whether the incidence of platform trials continued to increase despite a fading pandemic, the extent to which distinctive features were actually used, whether recruitment failures were rare, and whether results from platform trials were consistently made available. We, therefore, conducted a systematic review of all available randomized platform trials to empirically determine (1) their incidence over time, (2) the actual frequencies of various distinctive platform trial characteristics (eg, common control arm, use of nonconcurrent control data, and RAR), (3) the incidence of added and dropped arms over time, (4) the prevalence of discontinued trials due to poor participant recruitment, and (5) the availability of results for closed trial arms.

## Methods

This systematic review is reported according to the Preferred Reporting Items for Systematic Reviews and Meta-Analyses (PRISMA) reporting guideline.^26^ A detailed protocol was prospectively registered on Open Science Framework (OSF).^27^

### Systematic Search and Eligibility Criteria

The systematic search (including registries) was conducted on January 12, 2021, and was updated on July 28, 2022. Data were extracted until December 2022. Investigators were contacted for verification of the data in February 2023. We performed a systematic search of Medline (OVID), Embase (OVID), Scopus, and several trial registries (Clinicaltrials.gov, European Union Drug Regulating Authorities Clinical Trials Database, and International Standard Randomized Controlled Trial Number registry). To increase the sensitivity of the search, we included gray literature servers (OSF and Zenodo) and preprint servers (Europe PubMed Central) (search date: July 21, 2022). The detailed search strategy is available in eAppendix 1 in Supplement 1. An information specialist helped us design and review our search strategy. Trials were included if they were RCTs and planned to add or drop arms.

Screening of titles and abstracts, trial registries, and full text were performed in duplicate. Discrepancies were resolved by discussion or by involving a third reviewer (B.S. or M.B.). For each included report, we continued with forward and backward citation tracking (using Scopus). Citation tracking, gray literature, and preprint server screening was conducted by only 1 reviewer (A.G. or C.M.S.). If multiple reports were available for 1 platform trial, these reports were organized and consolidated by registry numbers, acronyms, and the title of the trial. Once a platform trial was included, we determined if an official trial website was available (by screening the literature and registries and searching via Google). For each platform trial and each of their recorded arms, we searched in duplicate (registry, website, Google Scholar, and Google) for the master protocol, subprotocols, and results publications, if not previously found in the literature search.

### Data Extraction

The variables for this systematic review were chosen based on discussions with methodologists and statisticians of platform trials, previous reviews on the topic, and the critical appraisal checklists by Park et al.^20,28^ All relevant data were extracted in duplicate (by different researchers). Differences were consolidated by a third reviewer. All authors worked in teams of 2 from trial protocols (master and subprotocols), result publications, trial registries, and the official trial websites into a REDCap data sheet.^29,30^ We documented the different labels used in study records (eg, “platform trial,” “trial platform,” “platform study,” “platform design,” or “platform protocol”) to explore the general use of the term *platform trial*. We extracted baseline characteristics for each included platform trial and each of their individual arms (see list of all baseline characteristics in eAppendix 2 in Supplement 1). Furthermore, distinct platform trial features were recorded. These features included the use of a common control arm and, if the common control arm could be updated during the trial, the use of nonconcurrent control data, adaptive design elements (eg, RAR, adaptive enrichment, seamless design, sample size readjustment), a statistical framework (bayesian, frequentist, or both), multiplicity adjustments (to multiple arms and for interim analyses), and feasibility studies (in silico trials or simulations or pilot trials). We determined the progression and output of the platform trial by the starting number of arms, the total number of arms, the number of arms added, the number of arms dropped (including the reason), and the status and availability of the results for each intervention arm (output of platform trial). Further features of interest included the use of biomarker stratification or subpopulations, integration of nonrandomized arms, interim analysis (reporting of frequency, outcome, and trigger), or the use of a factorial design. The format of the master protocol and the results publications were also recorded (as peer-reviewed publication, preprint, and full protocol on website or registry). Furthermore, we calculated the ratio of available results publications to the number of closed arms. The ratio was calculated twice, once including and once excluding results available as abstracts only. We contacted all principal investigators with a report detailing the most important information extracted from their platform trial. Principal investigators were asked to approve the accuracy of extracted data and to clarify missing or unclear information (eAppendix 3 in Supplement 1).

### Statistical Analysis

We summarized the characteristics of the included platform trials using the median and IQR for continuous variables and numbers and percentages for categorical variables. Baseline characteristics were stratified by sponsorship (industry vs not industry sponsored) and COVID-19 indication. Previous research has identified differences in the discontinuation rate, reporting quality, and transparency between industry-sponsored and non–industry-sponsored traditional RCTs^31,32^; as such, we stratified platform trial characteristics by sponsorship. Because it was expected that platform trial features are often recorded in the master protocol, we conducted a sensitivity analysis including only trials with an available master protocol. Data cleaning and analysis were conducted with R, version 1.4.1103 (R Project for Statistical Computing).

## Results

A total of 9155 records were identified. We determined 431 eligible records, resulting in 127 unique randomized platform trials included in our sample (the list of all included platform trials can be found in eTable 10 in Supplement 1). Labels such as “platform trial” and “platform study” were often used in a non–clinical trial context (see detailed list of all excluded reports using such terms in eTable 1 in Supplement 1). Platform trials were excluded if not randomized or if they did not allow for the adding and dropping of new arms (eFigure in Supplement 1).

Most platform trials were conducted in the fields of oncology (57 of 127 [44.9%]) and COVID-19 (45 of 127 [35.4%]), were multicenter and international (74 of 127 [58.3%]), tested drugs (108 of 127 [85.0%]), and were not industry sponsored (90 of 127 [70.9%]) (Table 1). All platform trials were registered. A master protocol was publicly available for 59.8% of all platform trials (76 of 127), and 16.5% (21 of 127) had also made older versions of protocols (amendments) available. A website existed for 51.2% of platform trials (65 of 127), with a higher prevalence observed in non–industry-sponsored trials than in industry-sponsored trials (55 of 90 [61.1%] vs 10 of 37 [27.0%]). Additional platform trial characteristics (eg, use of blinding, interim analyses, factorial design, nonrandomized arms, biomarker stratification, and number of subpopulations) and a stratification by COVID-19 and non–COVID-19 trials are presented in eTable 2, eTable 3, eTable 4, eTable 6, and eTable 7 in Supplement 1. A total of 38 platform trials (29.9%) were initiated in 2020, the highest reported incidence of newly started platform trials in 1 year thus far. This number has since decreased (25 of 127 [19.7%] in 2021) (Figure).

**Table 1.** General Platform Trial Characteristics

Characteristic	Trials, No. (%)		
	Industry sponsored (n = 37)	Not industry sponsored (n = 90)	Overall (N = 127)
Medical field			
Oncology	29 (78.4)	28 (31.1)	57 (44.9)
COVID-19	2 (5.4)	43 (47.8)	45 (35.4)
Other infectious diseases	1 (2.7)	8 (8.9)	9 (7.1)
Other^a^	5 (13.5)	11 (12.2)	16 (12.6)
Type of intervention			
Drug	36 (97.3)	72 (81.0)	108 (85.0)
Nondrug^b^	1 (2.7)	7 (7.8)	8 (6.3)
Both^b^	0	11 (12.2)	11 (8.7)
Trial phase			
Early	31 (83.8)	24 (26.7)	55 (43.3)
Late	3 (8.1)	44 (48.9)	47 (37.0)
Both	3 (8.1)	22 (24.4)	25 (19.7)
Planned sample size for platform trial			
<100	3 (8.1)	1 (1.1)	4 (3.1)
101-1000	29 (78.4)	36 (40.0)	65 (51.2)
>1000	2 (5.4)	39 (43.3)	41 (32.3)
Not reported	3 (8.1)	14 (15.6)	17 (13.4)
Funder			
Government or academic	0	57 (63.3)	57 (44.9)
Industry	35 (94.6)	1 (1.1)	36 (28.3)
Both	2 (5.4)	32 (36.6)	34 (26.8)
Multicenter or single center			
Single center	1 (2.7)	6 (7.7)	7 (5.5)
Multicenter and national	1 (2.7)	45 (50.0)	46 (36.2)
Multicenter and international	35 (94.6)	39 (43.3)	74 (58.3)
Trial registration	37 (100.0)	90 (100.0)	127 (100.0)
Protocol			
Master protocol			
Full protocol^c^	6 (16.2)	70 (77.8)	76 (59.8)
As poster or conference abstract	20 (54.1)	4 (4.4)	24 (18.9)
Not available	11 (29.7)	16 (17.8)	27 (21.3)
Old protocol versions (amendments) available	1 (2.7)	20 (22.2)	21 (16.5)
Website	10 (27.0)	55 (61.1)	65 (51.2)

^a^ Neurology (n = 6), dermatology (n = 2), general surgery (n = 2), gastrointestinal (n = 1), hematology (n = 1), nephrology (n = 1), diagnostic strategy (n = 1), genetic disease (n = 1), and respiratory (n = 1).

^b^ Vaccine (n = 7), surgical (n = 4), convalescent plasma (n = 1), dietary supplement (n = 1), mechanical ventilation (n = 1), radiotherapy (n = 2), medical device (n = 1), behavioral (n = 1), not defined (n = 1).

^c^ As peer-reviewed publication, on registry, as preprint or on website, or as letter to the editor.

**Figure.**
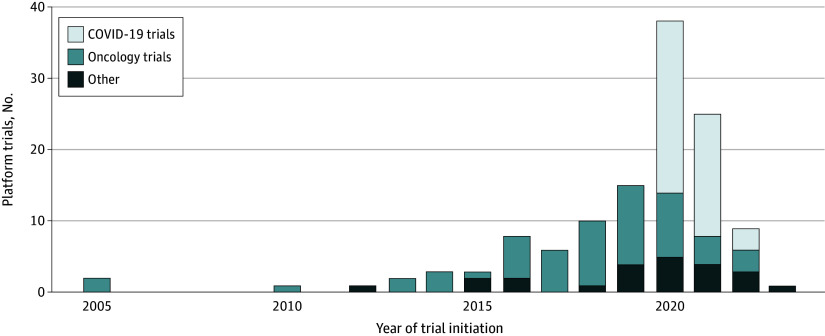
Frequency of Initiation of Platform Trials Our search ended in July 2022, and investigators were contacted to confirm their data accuracy in February 2023. The Figure includes 4 planned platform trials and the planned year of initiation.

A common control arm was reported to be used in 73.2% of all platform trials (93 of 127); 7.9% trials (10 of 127) planned to use nonconcurrent control data for their statistical analysis (not reported for 61 of 127 trials [48.0%]) (Table 2). Adaptive design elements were integrated in approximately half the platform trials (63 of 127 [49.6%]), and 17.3% of trials (22 of 127) implemented more than 1 adaptive design element. A correction for multiple testing for multiple arms was typically not reported (98 of 127 [77.2%]) or not considered (21 of 127 [16.5%]). The statistical framework was not reported by 37 studies (29.1%). Seamless designs, combining early- and late-phase trials, were used in 18.1% of trials (23 of 127). Characteristics stratified by COVID-19 vs non–COVID-19 trials can be found in eTable 4 in Supplement 1.

**Table 2.** Specific Platform Trial Characteristics

Characteristic	Trials, No. (%)		
	Industry sponsored (n = 37)	Not industry sponsored (n = 90)	Overall (N = 127)
Control group			
Common control used^a^	24 (64.9)	69 (76.7)	93 (73.2)
Nonconcurrent control			
Yes	0	10 (11.1)	10 (7.9)
No	7 (18.9)	49 (54.4)	56 (44.1)
Not reported	30 (81.1)	31 (34.4)	61 (48.0)
Planned duration			
Reported as perpetual	2 (5.4)	11 (12.2)	13 (10.2)
Fixed duration reported	35 (94.6)	75 (83.3)	110 (86.6)
Duration, median (IQR), mo	57.0 (39.5-71.8)	36.0 (24.0-66.0)	42.0 (24.0-68.0)
Not reported	0	4 (4.4)	4 (3.1)
Adaptive designs			
Additional adaptive design^b^^,^^c^	11 (29.7)	52 (57.8)	63 (49.6)
Response adaptive randomization	3 (8.1)	21 (23.3)	24 (18.9)
Sample size readjustment	1 (2.7)	18 (20.0)	19 (15.0)
Adaptive enrichment	0	10 (11.1)	10 (7.9)
Seamless	6 (6.7)	17 (18.9)	23 (18.1)
Adaptive dose adjustment	1 (2.7)	0	1 (0.8)
Statistical analysis			
Bayesian	5 (13.5)	38 (42.2)	43 (33.9)
Frequentists	5 (13.5)	34 (37.8)	39 (30.7)
Both	0	8 (8.9)	8 (6.3)
Not reported	27 (73.0)	10 (11.1)	37 (29.1)
Interim analyses reported	13 (35.1)	66 (73.3)	79 (62.2)
Multiple testing correction for multiple arms			
Corrected for multiple arms	0	8 (8.9)	8 (6.3)
No correction for multiple arms	1 (2.7)	20 (22.2)	21 (16.5)
Not reported	36 (97.3)	62 (68.9)	98 (77.2)
Trial feasibility assessment			
Feasibility or pilot study conducted	1 (2.7)	10 (11.1)	11 (8.7)
In silico trials (simulation conducted)	4 (10.8)	37 (41.1)	41 (32.3)

^a^ Common control planned to be updated (n = 31) or has been updated (n = 3) and cannot be updated (n = 8).

^b^ Multiple adaptive designs possible for platform trials.

^c^ Platform trials with 1 additional design (41 [65.1%]), 2 additional adaptive designs (17 [27.0%]), and 3 additional adaptive designs (5 [7.9%]).

Most randomized platform trials were ongoing (86 of 127 [67.7%]) or completed (26 of 127 [20.5%]), 4 of 127 (3.1%) were in planning, and 10 of 127 (7.9%) were discontinued (Table 3). Reasons for discontinuation included change in treatment landscape (3 of 10), low event rates (3 of 10), insufficient funding (2 of 10), and safety concerns (1 of 10), and, for 1 platform trial, the reason for discontinuation remained unclear. The number of arms at the start of the platform trial and the total number of arms was typically higher in industry-sponsored trials (median number of arms at start, 4 [IQR, 2-5]; median total number of arms, 6 [IQR, 4-8]) than in non–industry-sponsored trials (median number of arms at start, 3 [IQR, 2-4]; median total number of arms, 5 [IQR, 4-7]) (Table 3). Overall, 58.3% platform trials (74 of 127) added at least 1 arm, and 62.2% (79 of 127) dropped at least 1 arm during their progression; although planned, 21.3% of platform trials (27 of 127) neither added nor dropped an arm. Of the 85 platform trials that added or dropped an arm during the trial, the corresponding registry entry was not updated for 19 trials (22.4%). Half of all platform trials (64 of 127 [50.4%]) made results available from at least 1 comparison. Data on progression and output stratified by COVID-19 vs non–COVID-19 trials can be found in eTable 6 in Supplement 1.

**Table 3.** Platform Trial Progression and Output

Characteristic	Trials, No. (%)		
	Industry sponsored (n = 37)	Not industry sponsored (n = 90)	Overall (N = 127)
Status			
Ongoing	28 (75.7)	58 (64.4)	86 (67.7)
Completed	7 (18.9)	19 (21.1)	26 (20.5)
Discontinued^a^	2 (5.4)	8 (8.9)	10 (7.9)
In planning	0	4 (4.4)	4 (3.1)
Unclear	0	1 (1.1)	1 (0.8)
Adding and dropping of arms			
Starting No. of arms, median (IQR) [range]	4 (2 to 5) [2-10]	3 (2 to 4) [0-21]	3 (2-5) [0-21]
Total No. of arms, median (IQR) [range]	6 (4-8) [2-14]	5 (4-7) [0-52]	5 (4-8) [0-52]
Platform trials with added arms	29 (78.3)	45 (50.0)	74 (58.3)
No. of added arms, median (IQR)	2 (1-4)	1 (0-3)	1 (0-4)
Platform trials with dropped arms	20 (54.1)	59 (65.6)	79 (62.2)
No. of dropped arms, median (IQR)	1 (0-4)	2 (0-5)	2 (0-4)
Platform trials that neither added nor dropped arms^b^	3 (8.1)	24 (26.7)	27 (21.3)
Reporting of added or dropped arms			
Registry updated with added or dropped arms, No./total No. (%)^c^	28/30 (93.3)	38/55 (69.0)	66/85 (77.6)
Criteria reported for dropping and adding new arms, No./total No. (%)^d^	3/6 (50.0)	48/70 (68.6)	51/76 (67.1)
Results availability			
Any results available for platform trial	17 (45.9)	47 (52.2)	64 (50.4)

^a^ Reasons for discontinuation: change in treatment landscape (n = 3), low event rate (n = 3), insufficient funding (n = 2), safety (n = 1), and unclear (n = 1).

^b^ Includes the 4 planned platform trials.

^c^ Proportion calculated based on trials that added and dropped arms.

^d^ Proportion calculated based on trials with available master protocol.

The 127 platform trials had a total of 823 arms, including 206 control arms (Table 4). Of the 823 arms, 385 (46.8%) were ongoing, 34 (4.1%) were in the planning phase, and 353 (42.9%) were closed. Of the 353 closed arms, 189 (53.5%) were completed, 56 (15.9%) were stopped for futility, 20 (5.7%) were stopped due to new external evidence, 9 (2.5%) were stopped for safety concerns, and 26 (7.4%) were stopped for practical reasons, including poor recruitment (5 [1.4%]). Less than half of the closed arms (169 of 353 [47.9%]) made full results available. Making results available was more common and faster for non–industry-sponsored trials compared with industry-sponsored trials (150 of 277 [54.2%] vs 19 of 76 [25.0%]); however, there is evidence for confounding because COVID-19 trial results were available substantially faster than results for non–COVID-19 trials (Table 4). The detailed status of platform trial arms stratified by COVID-19 vs non–COVID-19 trials can be found in eTable 7 in Supplement 1. The form of results availability (as peer review, preprint, abstract, and on registry) is available in eTable 8 in Supplement 1. We contacted investigators of platform trials to verify the extracted data and achieved a high response rate (active agreement, 46.5% [59 of 127]; taciturn agreement, 15.7% [20 of 127]; no response, 37.8% [48 of 127]) (eTable 9 in Supplement 1).

**Table 4.** Status of Platform Trial Arms and Trial Arm Results

Characteristic	Arms, No./total No. (%)		
	Industry sponsored (n = 253)	Not industry sponsored (n = 570)	Overall (N = 823)
Total No. (%) of control arms reported	55 (21.7)	151 (26.5)	206 (25.0)
Status			
In planning	6 (2.4)	28 (4.9)	34 (4.1)
Ongoing	155 (61.3)	230 (40.4)	385 (46.8)
Closed	76 (30.0)	277 (48.6)	353 (42.9)
Arm reached target sample size^a^	43/76 (56.6)	146/277 (52.7)	189/353 (53.5)
Arm stopped due to futility	4/76 (5.3)	52/277 (18.8)	56/353 (15.9)
Arm stopped for safety	0/76 (0)	9/277 (3.2)	9/353 (2.5)
Arm stopped due to new external evidence	5/76 (6.6)	15/277 (5.4)	20/353 (5.7)
Arm stopped—reason for closure unclear	23/76 (30.3)	30/277 (10.8)	53/353 (15.0)
Arm stopped for practical reasons	1/76 (1.3)	25/277 (9.0)	26/353 (7.4)
Due to cessation of funding	1/1(100.0)	10/25 (40.0)	11/26 (42.3)
Due to recruitment problems	0/1 (0)	5/25 (20.0)	5/26 (19.2)
Due to low event rate	0/1 (0)	6/25 (24.0)	6/26 (23.1)
Due to operational problems	0/1 (0)	4/25 (16.0)	4/26 (15.4)
Suspended	0	3 (0.5)	3 (0.4)
Unclear	16 (6.3)	32 (5.6)	48 (5.8)
Published results for all closed arms (n = 353)			
Full results^b^	19/76 (25.0)	150/277 (54.2)	169/353 (47.9)
Time to results availability, median (IQR), d^c^	681.0 (531.0-1016.0)	219.0 (97.0-418.3)	227.0 (102.0-457.0)
COVID-19 trials (n = 95)	195.0 (97.0-242.5)	NA^d^	195.0 (97.0-242.5)
Non–COVID-19 trials (n = 74)	681.0 (671.0-1016.0)	472.0 (97.0-727.0)	554.5 (175.0-727.0)
Results including abstracts and press releases	38/76 (50.0)	192/277 (69.3)	230/353 (65.2)

Abbreviation: NA, not available.

^a^ Research question answered.

^b^ Full results as peer-reviewed results publications, preprints, and results entered into the registry; available for stopped arms.

^c^ Days from closure of arms plus follow-up to date of results availability.

^d^ No results available for this category at the time of this review.

## Discussion

Existing platform trials predominantly focus on evaluating drugs and tend to cluster in medical areas, such as oncology, COVID-19, and other infectious diseases. After the peak in 2020 with the arrival of the COVID-19 pandemic, the initiation of new platform trials has decreased. However, there has been a noticeable diversification of medical fields and interventions of platform trials over the past 5 years. This diversification encompasses areas such as neurology, dermatology, and general surgery, as well as the testing of behavioral, surgical, or dietary interventions.

Among the observed platform trials, 49.6% incorporated at least 1 additional adaptive design feature. A total of 58.3% of platform trials added at least 1 arm, and 62.2% dropped at least 1 arm (21.3% did neither, although planned). Consequently, the approximately 40% of trials that never added an arm may have incurred higher planning and setup costs compared with traditional RCTs without benefiting from the cost savings of additional arms.^33^ A common control arm was used in only 73.2% of platform trials, which is lower than one would expect for a major platform trial advantage (increased efficiency) and is below the percentage previously reported.^23^ This finding may underline the belief of many stakeholders that the establishment of collective trial infrastructures (including communication networks, overall data management and monitoring plans, and standardized documents across arms) is reason enough to justify the use of the platform trial design.^22^ Nevertheless, the benefits of only submitting an amendment instead of a new application for each added arm, and the quicker activation of sites, compared with new traditional RCTs, need to be balanced with substantial operational, statistical, and legal complexities of platform trials^21,34^

Many statistical features of platform trials are currently contended in literature, form the foundation of the platform trial design, and affirm the validity of the trial results.^12,16,22,35,36,37^ A bayesian design was frequently used because this statistical framework fits well with the adaptive nature of platform trials^25,35^; however, bayesian trial designs may be less commonly understood by a general medical and scientific readership, posing challenges for interpretation and uptake of results. In addition, the use of features such as RAR and nonconcurrent controls should be considered carefully. Response adaptive randomization, for instance, requires a well-planned run-in phase, may inflate type I error, typically requires a higher sample size, and can be associated with slow accrual of outcome data.^38^ About 8% of platform trials considered nonconcurrent control data in an attempt to further increase statistical power; however, this approach carries a high risk for bias.^22,37,39^ Regulators criticize the use of nonconcurrent controls in confirmatory trials because statistical modeling can only partially address the potential bias.^37,38^

Almost 80% of platform trial protocols were publicly available in some format, much higher than previously determined for traditional RCTs.^24,25^ However, reporting of essential features, such as adjustment for multiplicity, use of nonconcurrent control data, and criteria for dropping and adding new arms, was often unsatisfactory. Full results publications were available for 47.9% of closed arms. Premature closure of platform trial arms due to recruitment problems was infrequent, occurring in only 1.4% of trials, which is in contrast to traditional RCTs (discontinuation rate due to poor recruitment in RCTs, 10%-15%).^31,32^ However, it is possible that this proportion will increase due to recruitment hurdles and the increasing scarcity of eligible patients for COVID-19 trials toward the end of the pandemic. Publication of full results for closed arms (47.9%) was lower than what is generally seen for traditional RCTs (78.5% at 10-year follow-up).^32^ Availability of full results publications and overall transparency were generally better in non–industry-sponsored platform trials.

Overall, industry-sponsored platform trials accounted for approximately one-third of the total and predominantly focused on early-phase investigations, while late-phase trials were mostly not sponsored by industry. Seamless designs, combining early- and late-phase trials, although still a minority (18.1%), are becoming increasingly more common.^14^

### Strengths and Limitations

Our study has some strengths. To our knowledge, it is the first study investigating key platform trial features, protocol and results availability, and the status of individual arms. An additional strength of our study was that we contacted investigators of platform trials to verify the extracted data and achieved a high response rate (active agreement, 46.5% [59 of 127]; taciturn agreement, 15.7% [20 of 127]; no response, 37.8% [48 of 127]) (eTable 9 in Supplement 1); responses typically confirmed the accuracy of gathered data, and only minor adjustments were necessary.

Our study has the following limitations. First, available information was sometimes limited, especially if only a registry entry was available. We have, therefore, conducted sensitivity analyses showing how the proportion of certain variables changed if only platform trials with an available master protocol (n = 76 [59.8%]) were considered (eTable 5 in Supplement 1). Second, the reporting was not always consistent across different sources. We handled these discrepancies by creating an information hierarchy, giving priority to peer-reviewed manuscripts and the feedback received by investigators (followed by preprints, websites, and then other sources). Third, although highly desirable, we did not consider resource use and costs of platform trials in this review. Evidence from a hypothetical costing study suggested increased costs associated with the planning and setup of platform trials compared with traditional RCTs are due to the complex protocols and longer setup times.^33^ These increased costs were mitigated when more arms were added to the trial, which was less time intensive and reduced costs long term.^40,41^ Fourth, a comparison of platform trials with traditional parallel-arm RCTs was possible only on an indirect level. However, a direct comparison of platform trials with traditional RCTs with the same research question is planned in a future project, as described in our study protocol.^27^ Fifth, this systematic review provides only a snapshot of the current platform trial landscape. Two-thirds of identified platform trials are still ongoing, and the COVID-19 pandemic may have had an influence on the progression and output of our sample. Furthermore, methodological background and reporting guidelines for platform trials were lacking at the start of this project and are currently still evolving. Therefore, regular updates of this systematic review are necessary to gain further insights into progression patterns and output from randomized platform trials and to determine the most appropriate application of this design in the future.

## Conclusions

In this systematic review, we found that platform trials were initiated most frequently during the beginning of the COVID-19 pandemic and appeared to decrease thereafter, with a trend toward more diversified medical fields and interventions. Despite the potential for complexity, most made use of only 1 adaptive feature, or none. Forty percent of platform trials did not add an arm and, thereby, may have missed efficiency gains and incurred higher planning and setup costs compared with traditional RCTs.^33^ Premature arm closure for poor recruitment was rare. The reporting of platform features, the status of trial arms, and the results of closed arms needs to be improved. Guidance and infrastructure are needed so that the status and results of individual trial arms can be reported in a timely manner (eg, adaptations of trial registries for platform trials) and so that decisions about the need for a platform design and its planning is optimized.

## Notes

Supplement 1. **eAppendix 1.** Search Strategy **eAppendix 2.** Baseline Characteristics **eFigure 1.** Detailed Flow Chart and Reasons for Exclusion **eTable 1.** Report Labels and Reasons for Exclusion of Reports in Literature and Registry Screening **eTable 2.** Other Baseline Characteristics **eTable 3.** Baseline Characteristics by COVID and Non-COVID Platform Trials **eTable 4.** Specific Platform Trial Characteristics in COVID and Non-COVID Trials **eTable 5.** Specific Platform Trial Characteristics for Platform Trials With Full Available Master Protocol **eTable 6.** Platform Trial Progression and Output of COVID and Non-COVID Trials **eTable 7.** Status of Platform Trial Arms and Trial Arm Results in COVID and Non-COVID Trials **eTable 8.** How Were Results Made Available for Arms? **eTable 9.** Survey Response Rates **eAppendix 3.** Example of eMail Template and Report Sent to Platform Trial Teams **eTable 10.** List of Randomized Platform Trials

Supplement 2.
Data Sharing Statement
