# Supplementary material for: Characteristics, Progression, and Output of Randomized Platform Trials: A Systematic Review
Source: JAMA Netw Open. 2024 Mar 20;7(3):e243109. doi: 10.1001/jamanetworkopen.2024.3109 (PMC10955344; doi:10.1001/jamanetworkopen.2024.3109)
Supplement: Supplement 1. — eAppendix 1. Search Strategy eAppendix 2. Baseline Characteristics eFigure 1. Detailed Flow Chart and Reasons for Exclusion eTable 1. Report Labels and Reasons for Exclusion of Reports in Literature and Registry Screening eTable 2. Other Baseline Characteristics eTable 3. Baseline Characteristics by COVID and Non-COVID Platform Trials eTable 4. Specific Platform Trial Characteristics in COVID and Non-COVID Trials eTable 5. Specific Platform Trial Characteristics for Platform Trials With Full Available Master Protocol eTable 6. Platform Trial Progression and Output of COVID and Non-COVID Trials eTable 7. Status of Platform Trial Arms and Trial Arm Results in COVID and Non-COVID Trials eTable 8. How Were Results Made Available for Arms? eTable 9. Survey Response Rates eAppendix 3. Example of eMail Template and Report Sent to Platform Trial Teams eTable 10. List of Randomized Platform Trials [file jamanetwopen-e243109-s001.pdf]

Supplementary Online Content

Griessbach A, Schönenberger CM, Taji Heravi A, et al. Characteristics, progression, and output of randomized platform trials: a systematic review. *JAMA Netw Open.* 2024;7(3):e243109. doi:10.1001/jamanetworkopen.2024.3109

eAppendix 1. Search Strategy

eAppendix 2. Baseline Characteristics

eFigure. Detailed Flow Chart and Reasons for Exclusion

eTable 1. Report Labels and Reasons for Exclusion of Reports in Literature and Registry Screening

eTable 2. Other Baseline Characteristics

eTable 3. Baseline Characteristics by COVID and Non-COVID Platform Trials

eTable 4. Specific Platform Trial Characteristics in COVID and Non-COVID Trials

eTable 5. Specific Platform Trial Characteristics for Platform Trials With Full Available Master Protocol

eTable 6. Platform Trial Progression and Output of COVID and Non-COVID Trials

eTable 7. Status of Platform Trial Arms and Trial Arm Results in COVID and Non-COVID Trials

eTable 8. How Were Results Made Available for Arms?

eTable 9. Survey Response Rates

eAppendix 3. Example of eMail Template and Report Sent to Platform Trial Teams

eTable 10. List of Randomized Platform Trials

This supplementary material has been provided by the authors to give readers additional information about their work.

© 2024 Griessbach A et al. *JAMA Network Open.*

eAppendix 1. Search Strategy

Medline
(platform trial*.ti,ab. OR trial platform*.ti,ab. OR trials platform*.ti,ab. OR platform design*.ti,ab. OR platform stud*.ti,ab. OR study platform.ti,ab OR platform clinical trial*.ti,ab. OR clinical trials platform*.ti,ab. OR adaptive platform*.ti,ab. OR platform protocol*.ti,ab. OR platform randomized controlled*.ti,ab.) OR ((MAMS OR multi-arm multi* OR multi arm multi* OR umbrella trial* OR umbrella design*).ti,ab. ADJ8 (adaptive OR interim anal* OR flexible OR adjust*).ti,ab.)

Embase
(platform trial*.ti,ab. OR trial platform*.ti,ab. OR trials platform*.ti,ab. OR platform design*.ti,ab. OR platform stud*.ti,ab. OR study platform.ti,ab OR platform clinical trial*.ti,ab. OR clinical trials platform*.ti,ab. OR adaptive platform*.ti,ab. OR platform protocol*.ti,ab. OR platform randomized controlled*.ti,ab.) OR ((MAMS OR multi-arm multi* OR multi arm multi* OR umbrella trial* OR umbrella design*).ti,ab. ADJ8 (adaptive OR interim anal* OR flexible OR adjust*).ti,ab.)

Scopus
(TITLE-ABS("platform trial*") OR TITLE-ABS("trial platform*") OR TITLE-ABS("trials platform*") OR TITLE-ABS("platform design*") OR TITLE-ABS("platform stud*") OR TITLE-ABS("study platform") OR TITLE-ABS("platform clinical trial*") OR TITLE-ABS("clinical trials platform*") OR TITLE-ABS("adaptive platform*") OR TITLE-ABS("platform protocol*") OR TITLE-ABS("platform randomized controlled*")) OR ((TITLE-ABS("MAMS") OR TITLE-ABS("multi-arm multi*") OR TITLE-ABS("multi arm multi*") OR TITLE-ABS("umbrella trial*") OR TITLE-ABS("umbrella design*")) W/8 (TITLE-ABS("adaptive") OR TITLE-ABS("interim anal*") OR TITLE-ABS("flexible") OR TITLE-ABS("adjust*"))) AND (EXCLUDE (SUBJAREA , "ENGI") OR EXCLUDE (SUBJAREA , "COMP"))

Clinicaltrials.gov
"platform trial" OR "trial platform" OR "trials platform" OR "platform design" OR "platform study" OR "study platform" OR "platform clinical trial" OR "clinical trials platform" OR "adaptive platform" OR "platform protocol" OR "platform randomized controlled" OR

(("MAMS" OR "multi-arm multi" OR "multi arm multi" OR "umbrella trial" OR "umbrella design") AND ("adaptive" OR "interim analysis" OR "flexible OR adjust"))

EUDRA CT
"platform trial" OR "trial platform" OR "trials platform" OR "platform design" OR "platform study" OR "study platform" OR "platform clinical trial" OR "clinical trials platform" OR "adaptive platform" OR "platform protocol" OR "platform randomized controlled" OR

("MAMS" OR "multi-arm multi" OR "multi arm multi" OR "umbrella trial" OR "umbrella design") AND ("adaptive" OR "interim analysis" OR "flexible OR adjust")

ISRCTN
platform trial OR trial platform OR trials platform OR platform design OR platform study OR study platform OR platform clinical trial OR clinical trials platform OR adaptive platform OR platform protocol OR platform randomized controlled OR

((MAMS OR multi-arm multi OR multi arm multi OR umbrella trial OR umbrella design) AND (adaptive OR interim analysis OR flexible OR adjust))

ICRTP
"platform trial*" OR "trial platform" OR "trials platform" OR "platform design*" OR "platform stud*" OR "study platform" OR "platform clinical trial*" OR "clinical trials platform" OR "adaptive platform" OR "platform protocol" OR "platform randomized controlled"

("MAMS" OR "multi-arm multi*" OR "multi arm multi*" OR "umbrella trial*" OR "umbrella design") AND ("adaptive" OR "interim analysis")

© 2024 Griessbach A et al. *JAMA Network Open.*

eAppendix 2. Baseline Characteristics

We extracted the following baseline characteristics: study population, intervention arm(s), control arms(s), primary outcome, status of trial, medical field, type of intervention, late- or early-phase trial, sponsor (i.e. industry-sponsorship or non-industry sponsorship), funder (i.e. industry or non-industry), multi- or single-center, national or international, blinding (separately for patients, person applying intervention and outcome assessor) and use of a placebo. Early-phase trials were defined as phase 1-2 trials focusing on safety or pharmacodynamics and pharmacokinetics, whereas late-phase trials were defined as phase 3 and above. The status (in planning, ongoing, completed, discontinued/terminated/stopped, unclear), duration (start and end date), planned sample size and actual number of patients recruited were extracted for each platform trial as well as for each of the individual arms.

© 2024 Griessbach A et al. *JAMA Network Open.*

eFigure. Detailed Flow Chart and Reasons for Exclusion

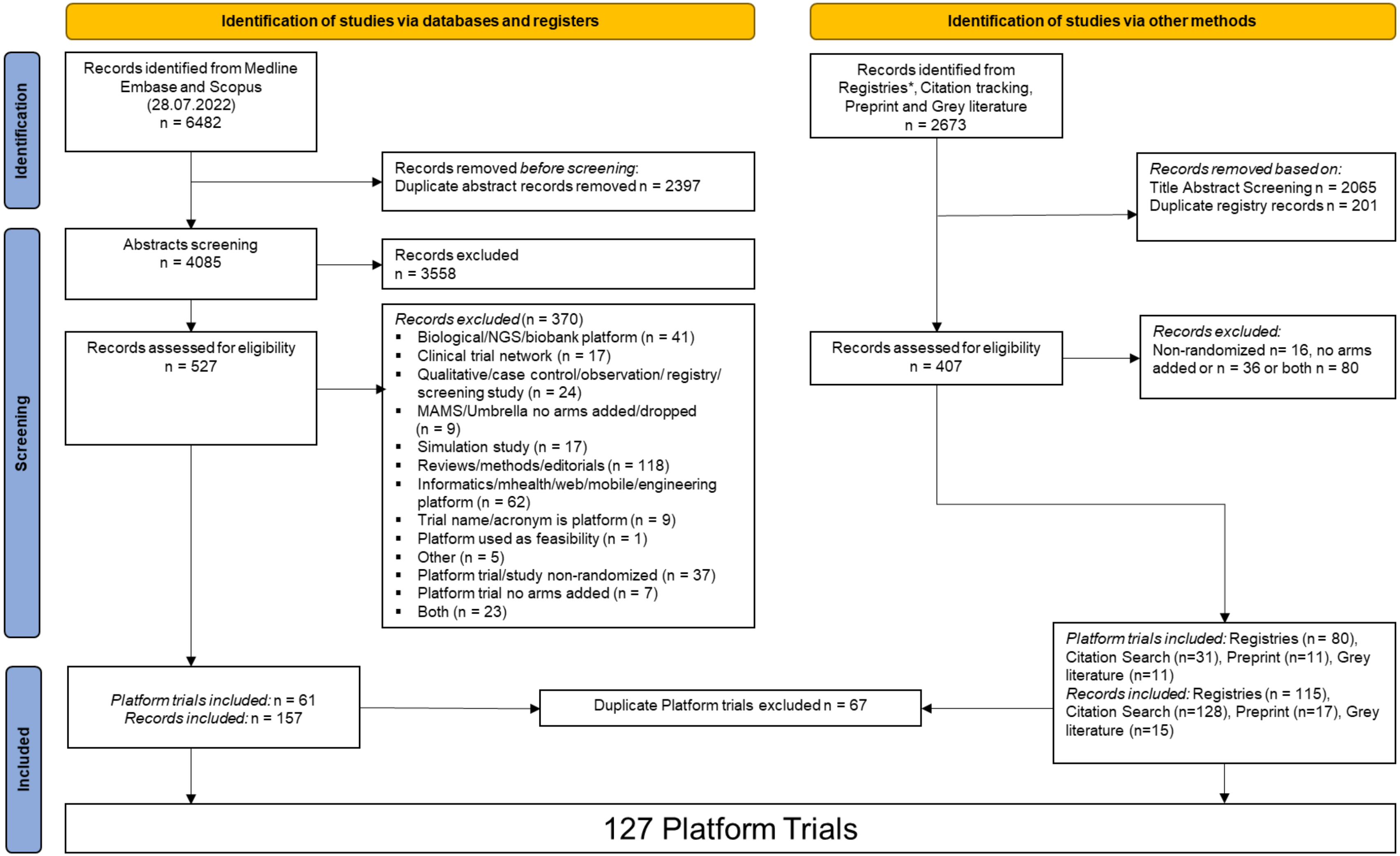

*Registries** Clinialtrials.gov (n=232), EUDRACT (n=61), ICTRP (n=116) and ISRCTN (n=73)

From: Page MJ, McKenzie JE, Bossuyt PM, Boutron I, Hoffmann TC, Mulrow CD, et al. The PRISMA 2020 statement: an updated guideline for reporting systematic reviews. BMJ 2021;372:n71. doi: 10.1136/bmj.n71. For more information, visit: http://www.prisma-statement.org/

© 2024 Griessbach A et al. *JAMA Network Open.*

eTable 1. Report Labels and Reasons for Exclusion of Reports in Literature and Registry Screening

Label	"platform trial"	"trial platform"	"platform study" or "study platform"	"platform design"	"novel platform"	"adaptive platform"	"platform protocol"
**Reasons for exclusion**							
Non-clinical trial related exclusion							
Biological platform, NGS platform or biological implant	10	3	13	18	-	3	2
Clinical trial network	3	18	10	2	-	-	-
Informatics/ mhealth/ mobile/engineering/ imaging platform	16	8	39	16	1	4	-
Clinical trial related exclusion reasons							
No arms added	18	1	14	-	-	-	-
PLATFORM as acronym	9	-	-	-	-	-	-
No randomization	19	1	35	7	-	-	1
No randomization or arms added	38	-	7	-	-	1	-
Report based exclusion							
Qualitative study /case control study/ observational study / registry study	12	-	22	2	-	-	1
Review/ commentary/ editorial/ methods	96	3	-	5	-	-	1
Simulation study	11	-	-	1	-	-	1
Other *	13	1	-	-	-	-	-

*platform used as term for "feasibility" (n=9); Reference to a platform trial (n= 4), Center or Site of trial has "clinical trial platform" in official name (n=1)

© 2024 Griessbach A et al. *JAMA Network Open.*

eTable 2. Other Baseline Characteristics

	Industry-sponsored (n=37)	Non-industry sponsored (n=90)	Overall (n=127)
**Blinding (%)**			
Patient blinded	6 (16.2)	28 (31.1)	34 (26.8)
Care giver blinded	5 (13.5)	24 (26.7)	29 (22.8)
Outcome assessor blinded	6 (16.2)	23 (25.6)	29 (22.8)
**Interim analyses (%)**			
Yes	13 (35.1)	66 (73.3)	79 (62.2)
Outcome reported	8 (61.5)	46 (69.7)	54 (68.4)
Number of interim analyses reported	7 (53.8)	32 (48.5)	39 (49.4)
Trigger reported [d]	10 (76.9)	52 (78.8)	62 (78.4)
Multiple testing adjustment due to interim analyses	0 (0.0)	13 (19.7)	13 (16.5)
**Other Design features (%)**			
Factorial	0 (0.0)	10 (11.1)	10 (7.8)
Non-randomized arms in platform trial	9 (24.3)	8 (8.9)	17 (13.4)
Biomarker stratification	7 (18.9)	19 (21.1)	26 (20.4)
Sub-populations	19 (51.4)	39 (43.3)	58 (45.7)
Median number of subpopulations	3 [2, 4]	3.00 [2, 4]	3 [2, 4]
Cross Over Design	0 (0.0)	1 (1.1)	1 (0.8)
**Placebo (%)** [a]	5 (13.5)	28 (31.1)	33 (26.0)

[a] Placebo used in at least one arm of the platform trial

© 2024 Griessbach A et al. *JAMA Network Open.*

eTable 3. Baseline Characteristics by COVID and Non-COVID Platform Trials

	COVID (n=45)	Non-COVID (n=82)	Overall (n=127)
**Status (%)**			
Ongoing [a]	27 (60.0)	59 (72.0)	86 (67.7)
Completed	13 (28.9)	13 (15.9)	26 (20.5)
Discontinued/terminated [b]	3 (6.7)	7 (8.5)	10 (7.9)
In planning	2 (4.4)	2 (2.4)	4 (3.1)
Unclear	0 (0.0)	1 (1.2)	1 (0.8)
**Sponsorship (%)**			
Industry	2 (4.4)	35 (42.7)	37 (29.1)
Non-Industry	43 (95.6)	47 (57.3)	90 (70.9)
**Type of intervention (%)**			
Drug	37 (82.2)	71 (86.6)	108 (85)
Non-drug [c]	6 (13.3)	2 (2.4)	8 (6.3)
Both [c]	2 (4.4)	9 (11.0)	11 (8.7)
**Trial Phase (%)**			
Early Phase Trial	8 (17.8)	47 (57.3)	55 (43.3)
Late Phase Trial	28 (62.2)	19 (23.1)	47 (37.0)
Both	9 (20.0)	16 (19.5)	25 (19.7)
**Planned sample size for platform trial (%)**			
<100	0 (2.1)	4 (4.9)	4 (3.1)
101-1000	12 (26.7)	53 (64.6)	65 (51.2)
>5000	25 (55.6)	16 (19.5)	41 (32.3)
Not reported	8 (17.8)	9 (11.0)	17 (13.4)
**Funder (%)**			
Public	34 (75.0)	23 (28.0)	57 (44.9)
Industry	2 (4.4)	34 (41.5)	36 (28.3)
Both	9 (20.0)	25 (30.5)	34 (26.8)
**Multi- or single center (%)**			
Single center	3 (6.7)	4 (4.9)	7 (5.5)
Multicenter- national	21 (46.7)	25 (30.5)	46 (36.2)
Multicenter- international	21 (46.7)	53 (64.6)	74 (58.3)
**Registration (%)**	45 (100.0)	78 (100)	127 (100)
**Protocol (%)**			
Master protocol			
Full protocol[d]	37 (82.2)	39 (47.6)	76 (59.3)
As poster or conference abstract	0 (0.0)	24 (29.3)	24 (18.9)
Not available	8 (17.8)	19 (23.2)	27 (21.3)
Old protocol versions (amendments) available	14 (31.2)	7 (8.9)	21 (16.5)

[a]Neurology (n=6), Dermatology (n=2), General surgery (n=2), Gastro/intestinal (n=1), Hematology (n=1), Nephrology (n=1), Diagnostic strategy (n=1), Genetic disease (n=1) and Respiratory (n=1)
[b] Vaccine (n=7), surgical (n=4), convalescent plasma (n=1), dietary supplement (n=1), mechanical ventilation (n=1), radiotherapy (n=2), behavioral (n=1), not defined (n=1)
[c] Government and academic funding
[d] As peer reviewed publication, on registry, as pre-print or on website and as letter to the editor (n=1)

© 2024 Griessbach A et al. *JAMA Network Open.*

eTable 4. Specific Platform Trial Characteristics in COVID and Non-COVID Trials

	COVID (n=45)	Non-COVID (n=82)	Overall (n=127)
**Control group (%)**			
Common control used	38 (84.4)	55 (67.1)	93 (74.4)
Non-concurrent control			
Yes	4 (8.9)	6 (7.3)	10 (7.9)
No	30 (66.7)	26 (31.7)	56 (44.1)
Not reported	11 (24.4)	50 (61.0)	61 (48.0)
**Planned duration (%)**			
Reported as perpetual	3 (6.7)	10 (12.2)	13 (10.2)
Fixed duration reported	41 (91.1)	69 (84.1)	110 (86.6)
Duration in months (median [IQR])	24.00 [16.00, 35.00]	64.00 [43.50, 83.00]	42.0[24.0 to 68.0]
Not reported	1 (2.2)	3 (3.7)	4 (3.1)
**Adaptive Designs (%)** [b]			
Additional adaptive design	24 (53.3)	39 (47.5)	63 (49.6)
RAR	8 (17.8)	16 (19.5)	24 (18.9)
Sample size readjustment	12 (26.7)	7 (8.5)	19 (15.0)
Adaptive enrichment	5 (11.1)	5 (6.1)	10 (7.9)
Seamless	7 (15.6)	16 (19.5)	23 (18.1)
Adaptive dose adjustment	0 (0.0)	1 (1.2)	1 (0.8)
**Statistical Analysis (%)**			
Bayesian	17 (37.8)	26 (31.7)	43 (33.9)
Frequentists	19 (42.2)	20 (24.4)	39 (30.7)
Both	6 (13.3)	2 (2.4)	8 (6.3)
Not reported	3 (6.7)	34 (41.5)	37 (29.1)
**Multiple testing correction for multiple arms (%)**			
Corrected for multiple arms	4 (8.8)	4 (4.8)	8 (6.3)
No correction for multiple arms	14 (31.8)	7 (8.5)	21 (16.5)
Not reported	27 (60.0)	71 (86.6)	98 (77.2)
**Trial Feasibility assessment (%)**			
Feasibility or pilot study conducted	4 (8.8)	7 (8.5)	11 (8.7)
In silico trials (simulation conducted)	18 (40.0)	23 (28.0)	41 (32.3)

© 2024 Griessbach A et al. *JAMA Network Open.*

eTable 5. Specific Platform Trial Characteristics for Platform Trials With Full Available Master Protocol

	Industry sponsored (n=6)	Non-industry sponsored (n=70)	Overall (n=76)
**Control group (%)**			
Common control used	6 (100.0)	58 (82.9)	64 (84.2)
Non-concurrent control			
Only concurrent control data	0 (0.0)	44 (62.9)	44 (58.0)
Use non-concurrent control data	0 (0.0)	8 (11.4)	8 (10.5)
Not reported	6 (100.0)	16 (22.9)	22 (28.9)
**Planned duration (%)**			
Reported as perpetual	1 (16.7)	11 (15.7)	12 (15.8)
Fixed duration reported	5 (83.3)	65 (85.5)	70 (92.1)
Duration in months (median [IQR])	72.0 [47.0 to 77.0]	36.0 [24.0 to 66.0]	42.0 [24.0 to 68.0]
**Adaptive Designs (%)** [b]			
Additional adaptive design	5 (83.3)	46 (65.7)	51 (67.1)
RAR	3 (50.0)	18 (25.7)	21 (27.6)
Sample size readjustment	0 (0.0)	16 (22.9)	16 (21.0)
Adaptive enrichment	0 (0.0)	9 (12.9)	9 (11.8)
Seamless	1 (0.0)	15 (21.4)	16 (21.0)
Adaptive dose adjustment	1 (33.3)	0 (0.0)	1 (1.3)
**Statistical Analysis (%)**			
Bayesian	2 (33.3)	31 (44.3)	33 (43.4)
Frequentists	1 (16.7)	30 (42.9)	31 (40.7)
Both	0 (0.0)	7 (10.0)	7 (9.2)
Not reported	3 (50.0)	2 (2.9)	5 (6.6)
**Multiple testing correction for multiple arms (%)**			
Corrected for multiple arms	0 (0.0)	8 (11.4)	8 (10.5)
No correction for multiple arms	0 (0.0)	16 (22.8)	16 (21.1)
Not reported	6 (100.0)	46 (65.7)	52 (68.4)
**Trial Feasibility assessment (%)**			
Feasibility or pilot study conducted	1 (16.7)	8 (11.4)	9 (11.8)
In silico trials (simulation conducted)	2 (33.3)	36 (51.4)	38 (50.0)

© 2024 Griessbach A et al. *JAMA Network Open.*

eTable 6. Platform Trial Progression and Output of COVID and Non-COVID Trials

	COVID (n=45)	Non-COVID (n=82)	Overall (n=127)
**Status**			
Ongoing	27 (60.0)	59 (72.9)	86 (67.7)
Completed	13 (28.9)	13 (15.9)	26 (20.5)
Discontinued [a]	3 (6.7)	7 (8.5)	10 (7.9)
In planning	2 (4.4)	2 (2.4)	4 (3.1)
Unclear	0 (0.0)	1 (1.2)	1 (0.8)
**Adding and dropping of arms**			
Start number of arms (median [IQR])	3.00 [2.00, 4.00]	4.00 [3.00, 5.00]	3 [2 to 5]
Range (min-max)	2-10	0-11	0-21
Total number of arms (median [IQR])	4.50 [3.00, 8.00]	6.00 [4.00, 8.00]	5 [4 to 8]
Range (min-max)	2-19	2-52	0-52
Platform trials with added arms (%)	26 (59.1)	48 (59.3)	74 (58.3)
Median number of added arms [IQR])	1.00 [0.00, 4.25]	1.00 [0.00, 4.00]	1 [0 to 4]
Platform trials with dropped arms (%)	37 (82.2)	42 (51.2)	79 (62.2)
Median number of dropped arms (median [IQR])	4.00 [2.00, 5.00]	1.00 [0.00, 3.00]	2 [0 to 4]
Platform trials that neither added nor dropped arms (%)[b]	7 (15.6)	20 (24.4)	27 (21.3)
**Reporting of added or dropped arms**			
Registry updated with added or dropped arms [c]	21 (46.7)	46 (56.1)	66/85 (77.6)
Criteria reported for dropping and adding new arms [d]	23/37 (62.2)	28/39 (45.9)	51/76 (67.1)
**Results availability**			
Any results available for platform trial	32 (71.1)	32 (39.0)	64 (50.4)

[a] Reasons for discontinuation: Change in treatment landscape (n=3), low event rate (n=3), insufficient funding (n=2), safety (n=1) and unclear (n=1)
[b] Includes the four planned platform trials
[c] Proportion calculated based on trials that have added and dropped arms
[d] Proportion calculated based on trials with available master protocol

© 2024 Griessbach A et al. *JAMA Network Open.*

eTable 7. Status of Platform Trial Arms and Trial Arm Results in COVID and Non-COVID Trials

	COVID (n=257)	Non-COVID (n=566)	Overall (n=823)
**Number of control arms**	64 (24.9)	142 (25.0)	206 (25.0)
**Status**			
In planning	6 (2.3)	28 (4.9)	34 (4.1)
Ongoing	77 (30.0)	308 (54.4)	385 (46.8)
Closed	166 (64.6)	187 (33.0)	353 (42.9)
Arm reached target sample size [a]	76/166 (45.8)	113/187 (60.4)	189/353 (53.5)
Arm stopped due to futility	35/166 (21.1)	21/187 (11.2)	56/353 (15.9)
Arm stopped for safety	1/166 (0.6)	8/187 (4.2)	9/353 (2.5)
Arm stopped due to new external evidence	13/166 (7.8)	7/187 (3.7)	20/353 (5.7)
Arm stopped, reason for closure unclear	26/166 (15.7)	27/187 (14.4)	53/353 (15.0)
Arm stopped for practical reasons	15/166 (9.0)	11/187 (5.9)	26/353 (7.4)
due to cessation of industry funding	0/15 (0.0)	11/11 (100.0)	11/26 (42.3)
due to recruitment problems	5/15 (30.0)	0/11 (0.0)	5/26 (19.2)
due to low event rate	6/15 (40.0)	0/11 (0.0)	6/26 (23.1)
due to operational problems	4/15 (26.7)	0/11 (0.0)	4/26 (15.4)
Suspended	3 (1.2)	0/11 (0.0)	3 (0.4)
Unclear	4 (1.6)	44 (7.8)	48 (5.8)
**Published results for all closed arms? (n=353)**			
Full results [b]	95/166 (57.2)	74/187 (39.6)	169/353 (47.9)
Time to results availability [c]	195.0 [97.0, 242.5]	554.5 [175.0, 727.0]	227.0 [102.0, 457.0]
Results including abstracts and press releases	112/166 (67.5)	118/187 (63.1)	230/353 (65.2)

[a] Research question answered
[b] Full results as peer reviewed results publications, preprints and results entered into the registry, available for stopped arms
[c] Days from closure of arms plus follow up to date of results availability

© 2024 Griessbach A et al. *JAMA Network Open.*

eTable 8. How Were Results Made Available for Arms?

	Industry sponsored (n=253)	Non-industry sponsored (n=570)	Overall (n=823)
**In what form are results available?** [a]			
As peer reviewed publication	6	146	152
As pre-print	0	45	45
Results available in trial registry	13	31	44
As conference abstract or poster	47	59	106
Other (e.g. press release)	8	65	73

© 2024 Griessbach A et al. *JAMA Network Open.*

eTable 9. Survey Response Rates

	n	%
Active response	59	46.5
Tacid agreement*	20	15.7
No Response	48	37.8

*if there were no unclear or open questions for study team, non-response was accepted as confirmation of data.

© 2024 Griessbach A et al. *JAMA Network Open.*

eAppendix 3. Example of eMail Template and Report Sent to Platform Trial Teams

Dear Dr. xxx/ Dear Sir or Madame,

We are a research group from the University Hospital Basel, Switzerland, conducting a systematic review on platform trials (study protocol registered at Open Science Framework: https://osf.io/epbcg). Our goal is to get an overview of all existing platform trials. We are contacting you because we identified a platform trial conducted by your company for which you are listed as the responsible person (i.e. "**XXXTITLEXXXX**"). Within the attached PDF we have listed the details which we have extracted for your platform trial. Please let us know if something is incorrect (e.g. status of platform trial incorrect, trial arm missing). In case we do not hear back from you, we will consider the current extractions as correct.

In addition we also have a few specific questions which we would like to ask you in order to better understand your platform trial.

Please feel free to forward this email if you feel that someone else could better answer the questions below:

STATUS OF PLATFORM TRIAL UNCLEAR

From available sources the current status of your platform trial is unclear. Is the overall platform trial still ongoing, completed, or discontinued (including reason for discontinuation)?

STATUS OF PLATFORM TRIAL INCONSISTENT WITH STATUS OF ARMS

From available sources we have seen that the status of your platform trial was "Ongoing", even though all treatment arms (with the exception of the control arm) seem to be closed. Could you please tell us if this is correct (i.e. platform trial is still open but without current active recruitment) or is it possible that one of those information is not up-to date?

MISSING PUBLICATIONS for completed arms:

Based on available data we came to the conclusion that the following arms are no longer recruiting:

-Arm A
-Arm B
-Arm C
Using a sensitive search strategy, we could not find published results for these intervention arms. Could you let us know if there are any complete published results for these arms?

STATUS OF ARM UNCLEAR:

Based on available data, we are unsure about the status of the following arms (see below).

-Arm A
-Arm B
-Arm C
Could you please let us know if these arms are still recruiting, closed (i.e. recruitment completed), or prematurely discontinued (including reason for discontinuation)? Please also let us know if there are published results for any of those arms.

DISCONTINUED ARMS:

© 2024 Griessbach A et al. *JAMA Network Open.*

Based on available information, the following arms of your trial were prematurely discontinued.

-Arm A
-Arm B
-Arm C
Could you please provide us with the reasons why those arms were discontinued?

ADDING/DROPING ARMS:

We have seen that your platform trial never made use of the feature of dropping or adding a new treatment arm. Is this correct?

ADDING/DROPPING ARMS COMPLETED PLATORMS:

We have seen that your platform trial has not dropped arms or added new arms. Is this correct? Could you tell us the reason why you did not use this specific feature of a platform trial?

MULTICENTRE INTERNATIONAL OR NATIONAL:

Your platform trial was set up as a multicenter platform trial. Could you please let us know if recruitment is planned in a single country (national) or in multiple countries (international)?

We thank you already in advance for your help and look forward to your feedback.

Please let us know if you wish to receive further information about our project.

Kind regards,

Benjamin Speich, PhD
Alexandra Griessbach, PhD candidate
Prof. Matthias Briel

© 2024 Griessbach A et al. *JAMA Network Open.*

Platform Trial Systematic Review

Dear investigator,

Thank you for reviewing the information of your platform trial for our systematic review. If any questions arise please do not hesitate to contact us at alexandranatacha.griessbach@usb.ch or benjamin.speich@usb.ch

Yours sincerely,

Dr. Benjamin Speich, PhD cand. Alexandra Griessbach and Prof. Matthias Briel

Platform Trial Details

Title: Randomised Controlled Trials to Assess Approved SARS-CoV-2 Vaccines in Immunocompromised Patients: A Master Protocol for the Set-up of a Swiss Cohorts Based Trial Platform

Acronym: COVERALL

Registry numbers:

Clinicaltrials.gov: NCT04805125

EUDRACT: not found

Other registries: not found

Trial Characteristics

platform trial status	ongoing
master protocol publicly available?	Yes
start number of arms in trial	2
total number arms in trial	2

- if start number of arms or total number of arms is 9999 - then value could not be identified. If applicable, please provide us with this value.

Arm Characteristics

arm name	is this the control?	status of arm	reason for discontinued/ unclear status	results available?
Moderna mRNA COVID-19 vaccine	No	completed/graduated	-	yes
Comirnaty® (Pfizer / BioNTech) mRNA COVID-19 vaccine	No	completed/graduated	-	yes

Specific Platform Trial Characteristics

	specific platform trial characteristics
response adaptive randomisation	not reported
sample size readjustment	not reported
adaptive enrichment	not reported
seamless design	not reported
use of non-concurrent control data?	Not Reported
frequentist or Bayesian framework	frequentist
adjustment for multiplicity for multiple arms?	not reported
simulations conducted?	not reported

© 2024 Griessbach A et al. *JAMA Network Open.*

**eTable 10.** List of Randomized Platform Trials	
ACCORD-2: A Multicentre, Seamless, Phase 2 Adaptive Randomisation Platform Study to Assess the Efficacy and Safety of Multiple Candidate Agents for the Treatment of COVID-19 in Hospitalised Patients	
**Acronym**	ACCORD2
**Registry Number**	2020-001736-95, ISRCTN57085639
**Master protocol**	Wilkinson, T., Dixon, R., Page, C. *et al.* ACCORD: A Multicentre, Seamless, Phase 2 Adaptive Randomisation Platform Study to Assess the Efficacy and Safety of Multiple Candidate Agents for the Treatment of COVID-19 in Hospitalised Patients: A structured summary of a study protocol for a randomised controlled trial.
**Results**	Not available
A Multicenter, Randomized, Active Controlled, Open Label, Platform Trial on the Efficacy and Safety of Experimental Therapeutics for Patients With COVID-19 (Caused by Infection With Severe Acute Respiratory Syndrome Coronavirus-2)	
**Acronym**	ACOVACT
**Registry Number**	NCT04351724, EUCTR2020-001302-30-AT
**Master protocol**	Not available
**Results**	Karolyi M., Pawelka E. et al. Camostat Mesylate Versus Lopinavir/Ritonavir in Hospitalized Patients With COVID-19—Results From a Randomized, Controlled, Open Label, Platform Trial (ACOVACT)<br>Karolyi M., Omid S. et al. High Dose Lopinavir/Ritonavir Does Not Lead to Sufficient Plasma Levels to Inhibit SARS-CoV-2 in Hospitalized Patients With COVID-19
Randomized Master Protocol for Immune Modulators for Treating COVID-19	
**Acronym**	ACTIV-1
**Registry Number**	NCT04593940
**Master protocol**	LaVange L., Adam J. S. et al. Accelerating COVID-19 Therapeutic Interventions and Vaccines (ACTIV): Designing Master Protocols for Evaluation of Candidate COVID-19 Therapeutics
**Results**	Emily R. Ko, Kevin J. Anstrom, Reynold A. Panettieri **Jr** et al. Abatacept for Treatment of Adults Hospitalized with Moderate or Severe Covid-19<br>Jane A. O'Halloran, Eyal Kedar, Kevin J. Anstrom et al. Infliximab for Treatment of Adults Hospitalized with Moderate or Severe Covid-19
Adaptive Platform Treatment Trial for Outpatients With COVID-19 (Adapt Out COVID)	
**Acronym**	ACTIV-2
**Registry Number**	NCT04518410
**Master protocol**	Not available
**Results**	Kara W. Chew, Carlee Moser, Eric S. Daar et al. Bamlanivimab reduces nasopharyngeal SARS-CoV-2 RNA levels but not symptom duration in non-hospitalized adults with COVID-19<br>Deatrick E. Statement—Large Clinical Trial Will Test Combination Monoclonal Antibody Therapy for Mild/Moderate COVID-19
A Multicenter, Adaptive, Randomized, Blinded Controlled Trial of the Safety and Efficacy of Investigational Therapeutics for Hospitalized Patients With COVID-19	
**Acronym**	ACTIV-3A
**Registry Number**	NCT04501978, 2020-003278-37
**Master protocol**	Murray D. D., Babiker G. A. et al. Design and implementation of an international, multi-arm, multi-stage platform master protocol for trials of novel SARS-CoV-2 antiviral agents: Therapeutics for Inpatients with COVID-19 (TICO/ACTIV-3)
**Results**	Lundgren JD, Grund B, Barkauskas CE, Holland TL, Gottlieb RL et al. A Neutralizing Monoclonal Antibody for Hospitalized Patients with Covid-19. Epub 2020 Dec 22
A Multicenter, Adaptive, Randomized, Blinded Controlled Trial of the Safety and Efficacy of Investigational Therapeutics for Hospitalized Patients With Acute Respiratory Distress Syndrome Associated With COVID-19	
**Acronym**	ACTIV-3B (TESICO)
**Registry Number**	NCT04843761
**Master protocol**	Not available
**Results**	Not available
A Multicenter, Adaptive, Randomized Controlled Platform Trial of the Safety and Efficacy of Antithrombotic Strategies in Hospitalized Adults With COVID-19	
**Acronym**	ACTIV-4A
**Registry Number**	NCT04505774, 2020-004285-19
**Master protocol**	Not available
**Results**	Berger S. J., Kornblith Z. L. et al Effect of P2Y12 Inhibitors on Survival Free of Organ Support Among Non–Critically Ill Hospitalized Patients With COVID-19A Randomized Clinical Trial<br>Massachusetts Medical Society, Therapeutic Anticoagulation with Heparin in Critically Ill Patients with Covid-19<br>Massachusetts Medical Society, Therapeutic Anticoagulation with Heparin in Noncritically Ill Patients with Covid-19
COVID-19 Outpatient Thrombosis Prevention Trial: A Multi-center Adaptive Randomized Placebo-controlled Platform Trial Evaluating the Efficacy and Safety of Anti-thrombotic Strategies in COVID-19 Adults Not Requiring Hospitalization at Time of Diagnosis	
**Acronym**	ACTIV-4B
**Registry Number**	NCT04498273

© 2024 Griessbach A et al. *JAMA Network Open.*

**Master protocol**	Not available
**Results**	Connors M. J, Brooks M. M. et al. Effect of Antithrombotic Therapy on Clinical Outcomes in Outpatients With Clinically Stable Symptomatic COVID-19 The ACTIV-4B Randomized Clinical Trial
COVID-19 Post-hospital Thrombosis Prevention Trial: An Adaptive, Multicenter, Prospective, Randomized Platform Trial Evaluating the Efficacy and Safety of Antithrombotic Strategies in Patients With COVID-19 Following Hospital Discharge	
**Acronym**	ACTIV-4C
**Registry Number**	NCT04650087
**Master protocol**	Not available
**Results**	Not available
A Multicenter Platform Trial of Putative Therapeutics for the Treatment of COVID-19 in Hospitalized Adults	
**Acronym**	ACTIV-5
**Registry Number**	NCT04583956, NCT04583969, NCT04988035
**Master protocol**	Not available
**Results**	Temesgen Z., Kenderian S. S. et al. In Reply — Clinical Benefit of Lenzilumab in Cases of Coronavirus Disease 2019
A Multicenter, Adaptive, Randomized Blinded Controlled Trial of the Safety and Efficacy of Investigational Therapeutics for the Treatment of COVID-19 in Hospitalized Adults (ACTT)	
**Acronym**	ACTT
**Registry Number**	NCT04280705, NCT04401579, NCT04492475, NCT04640168
**Master protocol**	ClinicalTrial.gov, A Multicenter, Adaptive, Randomized Blinded Controlled Trial of the Safety and Efficacy of Investigational Therapeutics for the Treatment of COVID-19 in Hospitalized Adults
**Results**	Beigel H. J., Tomashek M. K. et al. Remdesivir for the Treatment of Covid-19 — Final Report<br>Kalil C. A., Patterson F. T. et al. Baricitinib plus Remdesivir for Hospitalized Adults with Covid-19<br>Kalil C. A., Mehta K. A. et al. Efficacy of interferon beta-1a plus remdesivir compared with remdesivir alone in hospitalised adults with COVID-19: a double-blind, randomised, placebo-controlled, phase 3 trial<br>Wolfe CR et al.,Baricitinib versus dexamethasone for adults hospitalised with COVID-19 (ACTT-4): a randomised, double-blind, double placebo-controlled trial. Lancet Respir Med. 2022 Sep;10(9):888-899. doi: 10.1016/S2213-2600(22)00088-1. Epub 2022 May 23.<br>National Institute of Allergy and Infectious Diseases BULLETIN—NIAID Stops Enrollment of Severely Ill COVID-19 Participants in Clinical Trial of Investigational Treatments (https://www.niaid.nih.gov/news-events/bulletin-niaid-stops-enrollment-severely-ill-covid-19-participants-clinical-trial)<br>NIH closes enrollment in trial comparing COVID-19 treatment regimens (https://www.nih.gov/news-events/news-releases/nih-closes-enrollment-trial-comparing-covid-19-treatment-regimens)
A randomized, open-label, phase I/II open platform study evaluating safety and efficacy of novel ruxolitinib combinations in myelofibrosis patients	
**Acronym**	ADORE
**Registry Number**	NCT04097821, 2019-000373-23
**Master protocol**	Ross M. D., Heidel H. F. et al. ADORE: A Randomized Open-Label, Phase 1/2, Open-Platform Study Evaluating Safety and Efficacy of Novel Ruxolitinib Combinations in Patients with Myelofibrosis<br>Perkins C. A., Burbury K. et al. Adore: A Randomized, Open-Label, Phase 1/2 Open-Platform Study Evaluating Safety and Efficacy of Novel Ruxolitinib Combinations in Patients with Myelofibrosis
**Results**	Not available
A study to assess several different treatments that may be useful for patients with COVID-19	
**Acronym**	AGILE-ACCORD
**Registry Number**	NCT04746183, 2020-001860-27, ISRCTN27106947
**Master protocol**	Griffiths O. G., FitzGerald R. et al. AGILE: a seamless phase I/IIa platform for the rapid evaluation of candidates for COVID-19 treatment: an update to the structured summary of a study protocol for a randomised platform trial letter
**Results**	Khoo H. S., Fitzgerald R. et al. Optimal dose and safety of molnupiravir in patients with early SARS-CoV-2: a Phase I, open-label, dose-escalating, randomized controlled study
Adjuvant Lung Cancer Enrichment Marker Identification and Sequencing Trial (ALCHEMIST)	
**Acronym**	ALCHEMIST
**Registry Number**	NCT02194738, NCT02193282, NCT02201992, NCT02595944, NCT04267848
**Master protocol**	Künkele A., Taraseviciute A. et al. Preclinical Assessment of CD171-Directed CAR T-cell Adoptive Therapy for Childhood Neuroblastoma: CE7 Epitope Target Safety and Product Manufacturing Feasibility<br>Alden S. R., Mandrekar J. S., Oxnard R. G. Designing a definitive trial for adjuvant targeted therapy in genotype defined lung cancer: the ALCHEMIST trials
**Results**	Not available
Antivirals for influenza-Like Illness? A randomised Controlled trial of Clinical and Cost effectiveness in primary CarE (ALIC4 E): the ALIC4 E protocol	
**Acronym**	ALIC4 E
**Registry Number**	2014-004471-23, ISRCTN27908921

© 2024 Griessbach A et al. *JAMA Network Open.*

**Master protocol**	Butler C. C., Coenen S. et al. A trial like ALIC^{4}E: why design a platform, response-adaptive, open, randomised controlled trial of antivirals for influenza-like illness?<br>Bongard E., Cook J. et al. Antivirals for influenza-Like Illness? A randomised Controlled trial of Clinical and Cost effectiveness in primary CarE (ALIC^4 E): the ALIC^4 E protocol
**Results**	Butler C. C., Bongard E., et al. Oseltamivir plus usual care versus usual care for influenza-like illness in primary care: an open-label, pragmatic, randomised controlled trial
A Phase 1b/2, Open-Label, Randomized Platform Study Evaluating the Efficacy and Safety of AB928-Based Treatment Combinations in Patients With Metastatic Castrate Resistant Prostate Cancer	
**Acronym**	ARC6
**Registry Number**	NCT04381832
**Master protocol**	ARC-6: A Phase 1b/2, Open-Label, Randomized Platform Study Evaluating the Efficacy and Safety of Etrumadenant (AB928)-Based Treatment Combinations in Patients with Metastatic Castrate-Resistant Prostate Cancer<br>SK Subudhi1, DR Wise2, ST Liu3, A Chaudhry4, J Kim5, OS Gardner6, HN Gilbert6, M Grady6, C Trudeau6, MC Paoloni6, K Krishnan6, and MA Carducci7
**Results**	Bendell C. J., Carducci A. M., et al. ARC-6: A phase 1b/2, open-label, randomized platform study to evaluate efficacy and safety of etrumadenant (AB928)-based treatment combinations in patients with metastatic castrate-resistant prostate cancer (mCRPC).
A Phase 1b/2, Open-Label, Randomized Platform Study Evaluating The Efficacy and Safety of AB928 Based Treatment Combinations in Patients With Metastatic Colorectal Cancer	
**Acronym**	ARC9
**Registry Number**	NCT04660812
**Master protocol**	Cecchini M., Krishnan K. et al. ARC-9: Phase 1b/2 Study to Evaluate Etrumadenant (AB928)-Based Treatment Combinations<br>in Patients with Metastatic Colorectal Cancer
**Results**	Not available
Antithrombotic Therapy to Ameliorate Complications of COVID-19 (ATTACC), in Collaboration With Accelerating COVID-19 Therapeutic Interventions and Vaccines (ACTIV-4)	
**Acronym**	ATTACC
**Registry Number**	NCT04372589
**Master protocol**	Houston L. B., Lawler R. P. et al. Anti-Thrombotic Therapy to Ameliorate Complications of COVID-19 (ATTACC): Study design and methodology for an international, adaptive Bayesian randomized controlled trial<br>https://www.nejm.org/doi/suppl/10.1056/NEJMoa2103417/suppl_file/nejmoa2103417_protocol.pdf University of Manitoba, AntiThrombotic Therapy to Ameliorate Complications of COVID-19 (ATTACC) in collaboration with Accelerating COVID-19 Therapeutic Interventions and Vaccines (ACTIV-4)
**Results**	Massachusetts Medical Society Therapeutic Anticoagulation with Heparin in Noncritically Ill Patients with Covid-19<br>Lawler R. P., Goligher C. E., et al. Therapeutic Anticoagulation in Non-Critically Ill Patients with Covid-19
Efficient adaptive randomised trial design in neuroblastoma: The beacon-neuroblastoma trial	
**Acronym**	BEACON
**Registry Number**	NCT02308527, 2012-000072-42, ISRCTN40708286
**Master protocol**	Moreno L., Laidler J. et al. A randomised phase IIb trial of BEvACizumab added to Temozolomide ± IrinOtecan for children with refractory/relapsed Neuroblastoma - BEACON-Neuroblastoma, a European Innovative Therapies for Children with Cancer (ITCC) - International Society of Paediatric Oncology Europe Neuroblastoma Group (SIOPEN) trial.
**Results**	Moreno L., Moroz V. et al. Temozolomide versus irinotecan-temozolomide for children with relapsed and refractory high risk neuroblastoma (RR-HRNB): Results of the BEACON-Neuroblastoma randomized phase 2 trial—A European Innovative Therapies for Children with Cancer (ITCC) - International Society of Pediatric Oncology Europe Neuroblastoma Group (SIOPEN) trial.<br>Moreno L., Moroz V., et al. LBA64 - Bevacizumab for children with relapsed & refractory high-risk neuroblastoma (RR-HRNB): Results of the BEACON-neuroblastoma randomized phase II trial - A European ITCC-SIOPEN trial<br>Moreno L., Laidler J. et al. A randomised phase IIb trial of BEvACizumab added to Temozolomide ± IrinOtecan for children with refractory/relapsed Neuroblastoma - BEACON-Neuroblastoma, a European Innovative Therapies for Children with Cancer (ITCC) - International Society of Paediatric Oncology Europe Neuroblastoma Group (SIOPEN) trial.<br>Moreno L., Moroz V. et al. Bevacizumab for children with relapsed & refractory high-risk neuroblastoma (RR-HRNB): Results of the BEACON-neuroblastoma randomized phase II trial - A European ITCC-SIOPEN trial
BEGONIA: Phase Ib/II open-label, platform study of safety and efficacy of durvalumab, paclitaxel and other novel oncology therapy agents as first-line (1L) therapy in patients with metastatic triple negative breast cancer (mTNBC)	
**Acronym**	BEGONIA: Phase Ib/II open-label, platform study of safety and efficacy of durvalumab, paclitaxel and other novel oncology therapy agents as first-line (1L) therapy in patients with metastatic triple negative breast cancer (mTNBC)
**Registry Number**	NCT03742102, 2018-000764-29

© 2024 Griessbach A et al. *JAMA Network Open.*

**Master protocol**	Schmid P., Dry H. et al. BEGONIA: Phase 1b/2, open-label, platform study of the safety and efficacy of durvalumab (D) ± paclitaxel (P) with novel oncology therapies for first-line metastatic triple-negative breast cancer (mTNBC): Addition of arm 7, D + datopotamab deruxtecan (Dato-DXd; DS-1062).<br>Schmid P., Nunes T. A. et al. Abstract OT3-01-01: BEGONIA: Phase Ib/II open-label, platform study of safety and efficacy of durvalumab, paclitaxel and other novel oncology therapy agents as first-line (1L) therapy in patients with metastatic triple negative breast cancer (mTNBC)
**Results**	Schmid P., Seock-Ah I. et al. BEGONIA: Phase 1b/2 study of durvalumab (D) combinations in locally advanced/metastatic triple-negative breast cancer (TNBC)—Initial results from arm 1, d+paclitaxel (P), and arm 6, d+trastuzumab deruxtecan (T-DXd).<br>Schmid P., Seock-Ah I. et al. BEGONIA: Phase 1b/2 study of durvalumab (D) combinations in locally advanced/metastatic triple-negative breast cancer (TNBC)—Initial results from arm 1, d+paclitaxel (P), and arm 6, d+trastuzumab deruxtecan (T-DXd)
An Open-Label, Randomised, Multi-Drug, Biomarker-Directed, Multi-Centre, Multi-arm Phase 1b Study in Patients With Muscle Invasive Bladder Cancer (MIBC) Who Have Progressed on Prior Treatment (BISCAY).	
**Acronym**	BISCAY
**Registry Number**	NCT02546661
**Master protocol**	Powler T., Kilgour E. et al. BISCAY, a phase Ib, biomarker-directed multidrug umbrella study in patients with metastatic bladder cancer.
**Results**	Powles T., Carroll D. et al. An adaptive, biomarker-directed platform study of durvalumab in combination with targeted therapies in advanced urothelial cancer
CATALYST - A randomised phase II proof of principle multi-arm multi-stage trial designed to guide the selection of interventions for phase III trials in hospitalised patients with COVID-19 infection.	
**Acronym**	CATALYST
**Registry Number**	2020-001684-89, ISRCTN40580903
**Master protocol**	Veenith T., Fisher A. B. et al. CATALYST trial protocol: a multicentre, open-label, phase II, multiarm trial for an early and accelerated evaluation of the potential treatments for COVID-19 in hospitalised adults
**Results**	Veenith T., Fisher A. B. et al. Namilumab or infliximab compared to standard of care in hospitalised patients with COVID-19 (CATALYST): a phase 2 randomised adaptive trial<br>Veenith T., Fisher A. B. et al. Namilumab or infliximab compared with standard of care in hospitalised patients with COVID-19 (CATALYST): a randomised, multicentre, multi-arm, multistage, open-label, adaptive, phase 2, proof-of-concept trial
Efficacy and safety of novel treatment options for adults with COVID-19 pneumonia. A double-blinded, randomized, multi-stage, 6-armed placebo-controlled trial in the framework of an adaptive trial platform	
**Acronym**	CCAP
**Registry Number**	NCT04345289, 2020-001367-88
**Master protocol**	Not available
**Results**	Thorlacius-Ussing L., Brooks T. P. et al. A randomized placebo-controlled trial of convalescent plasma for adults hospitalized with COVID-19 pneumonia
Imperial Prostate 4: Comparative Health Research Outcomes of NOvel Surgery in Prostate Cancer	
**Acronym**	CHRONOS
**Registry Number**	NCT04049747, 2019-001365-32, ISRCTN17796995
**Master protocol**	Reddy D. Shah T. T. et al. Comparative Healthcare Research Outcomes of Novel Surgery in prostate cancer (IP4-CHRONOS): A prospective, multi-centre therapeutic phase II parallel Randomised Control Trial<br>Day E., Prevost T. A. et al. Feasibility of Comparative Health Research Outcome of Novel Surgery in prostate cancer (IP4-CHRONOS): statistical analysis plan for the randomised feasibility phase of the CHRONOS study<br>Guillaumier S., Peters M. et al. A Multicentre Study of 5-year Outcomes Following Focal Therapy in Treating Clinically Significant Nonmetastatic Prostate Cancer<br>Shah T. T., Peters M. et al. Early-Medium-Term Outcomes of Primary Focal Cryotherapy to Treat Nonmetastatic Clinically Significant Prostate Cancer from a Prospective Multicentre Registry
**Results**	Not available
A Phase 2, Open-label, Multicenter, Randomized, Multidrug Platform Study of Durvalumab Alone or in Combination with Novel Agents in Subjects with Locally Advanced, Unresectable, Stage III Non-small Cell Lung Cancer (COAST)	
**Acronym**	COAST
**Registry Number**	NCT03822351, 2018-002931-35
**Master protocol**	R. Herbst et al. P1.04-28 COAST: Durvalumab Alone or with Novel Agents for Locally Advanced, Unresectable, Stage III Non-Small Cell Lung Cancer, Journal of Thoracic Oncology
**Results**	Martinez-Marti A., Majem M. et al. LBA42 COAST: An open-label, randomised, phase II platform study of durvalumab alone or in combination with novel agents in patients with locally advanced, unresectable, stage III NSCLC<br>Herbst S. R., Majem M. et al. COAST: An Open-Label, Phase II, Multidrug Platform Study of Durvalumab Alone or in Combination With Oleclumab or Monalizumab in Patients With Unresectable, Stage III Non–Small-Cell Lung Cancer
A Phase 1b/2, Open-label, Multicenter Study of Novel Oncology Therapies in Combination With Chemotherapy and Bevacizumab as First-line Therapy in Metastatic Microsatellite-stable Colorectal Cancer (COLUMBIA-1)	

© 2024 Griessbach A et al. *JAMA Network Open.*

**Acronym**	COLUMBIA-1
**Registry Number**	NCT04068610
**Master protocol**	Not available
**Results**	Not available
A Phase 2, Open-label, Randomized, Multicenter, Platform Study of Novel Oncology Therapies in Combination With Adjuvant Chemotherapy in High-risk, Microsatellite-stable Colorectal Cancer (COLUMBIA-2)	
**Acronym**	COLUMBIA-2
**Registry Number**	NCT04145193
**Master protocol**	Not available
**Results**	Not available
CONCORDE: A phase I platform study of novel agents in combination with conventional radiotherapy in non-small-cell lung cancer	
**Acronym**	CONCORDE
**Registry Number**	NCT04550104, 2020-000206-28, ISRCTN10142971
**Master protocol**	Walls M. G., Oughton B. J. et al. CONCORDE: A phase I platform study of novel agents in combination with conventional radiotherapy in non-small-cell lung cancer<br>Faivre-Finn C., Brown S. et al. The UK at the Forefront of Innovative Drug–Radiotherapy Combination Clinical Trials: Introducing the CONCORDE Platform
**Results**	Not available
COVID-19 Outpatient Pragmatic Platform Study (COPPS): A Pragmatic Multi-arm, Adaptive, Phase 2, Blinded, Randomized Placebo-controlled Platform Trial to Assess the Efficacy of Different Investigational Therapeutics in Reducing Time to Disease Resolution or Viral Load Cessation, as Compared to Standard Supportive Care in Outpatients With COVID-19	
**Acronym**	COPPS
**Registry Number**	NCT04662086, NCT04662073, NCT04662060
**Master protocol**	Bunning B., Hedlin H. et al. The COVID-19 Outpatient Pragmatic Platform Study (COPPS): Study design of a multi-center pragmatic platform trial
**Results**	Not available
The DIAN-TU Next Generation Alzheimer's prevention trial: Adaptive design and disease progression model	
**Acronym**	DIAN-TU
**Registry Number**	NCT01760005, NCT04623242 (results submitted not posted), 2013-000307-17
**Master protocol**	Bateman J. R., Benzinger L. T. et al. The DIAN-TU Next Generation Alzheimer's prevention trial: Adaptive design and disease progression model<br>Bateman J. R., Aschenbrenner J. A. et al. Overview of dominantly inherited AD and top-line DIAN-TU results of solanezumab and gantenerumab
**Results**	Not available
Antiviral drugs in hospitalized patients with COVID-19 - the DisCoVeRy trial	
**Acronym**	DISCOVERY
**Registry Number**	NCT04315948, 2020-000936-23
**Master protocol**	Ader F. Protocol for the DisCoVeRy trial: multicentre, adaptive, randomised trial of the safety and efficacy of treatments for COVID-19 in hospitalised adults
**Results**	Ader F., Hites M. et al. Remdesivir plus standard of care versus standard of care alone for the treatment of patients admitted to hospital with COVID-19 (DisCoVeRy): a phase 3, randomised, controlled, open-label trial<br>Ader F., Poissy J. et al. Antiviral drugs in hospitalized patients with COVID-19 - the DisCoVeRy trial<br>Ader F., Poissy J. et al. An open-label randomized controlled trial of the effect of lopinavir/ritonavir, lopinavir/ritonavir plus IFN-β-1a and hydroxychloroquine in hospitalized patients with COVID-19
A Phase I/II, Randomized, Open-label Platform Study Utilizing a Master Protocol to Study GSK2857916 as Monotherapy and in Combination with Anti-Cancer Treatments in Participants with Relapsed/Refractory Multiple Myeloma (RRMM)	
**Acronym**	DREAMM5
**Registry Number**	NCT04126200, 2019-001138-32
**Master protocol**	Nooka K. A., Weisel K. et al. Belantamab mafodotin in combination with novel agents in relapsed/refractory multiple myeloma: DREAMM-5 study design<br>Richardson G. P, Trudel S. et al. P-212: DREAMM-5 platform trial: Belantamab mafodotin (belamaf; GSK2857916) in combination with five different novel agents in patients with relapsed/refractory multiple myeloma (RRMM)<br>Richardson G. P., Nooka A. et al. Dreamm-5 Platform Trial: Belantamab Mafodotin (Belamaf) in Combination with Four Different Novel Agents in Patients with Relapsed/Refractory Multiple Myeloma (RRMM)
**Results**	Lonial S., Grosicki S. et al. Synergistic effects of low-dose belantamab mafodotin in combination with a gamma-secretase inhibitor (nirogacestat) in patients with relapsed/refractory multiple myeloma (RRMM): DREAMM-5 study.
An Adaptive Randomized Trial Comparing Multiple Treatments for Ebola Virus (EBOV) Infected Children and Adults	
**Acronym**	EVD-003
**Registry Number**	NCT02380625
**Master protocol**	Berry M. S., Petzhold A. E. et al. A response adaptive randomization platform trial for efficient evaluation of Ebola virus treatments: A model for pandemic response
**Results**	Not available
European Prevention of Alzheimer's Dementia (EPAD) Longitudinal Cohort Study (LCS) (EPAD-LCS)	

© 2024 Griessbach A et al. *JAMA Network Open.*

**Acronym**	EPAD
**Registry Number**	NCT02804789
**Master protocol**	Not available
**Results**	Not available
European DisCoVeRy for Solidarity: An Adaptive Pandemic and Emerging Infection Platform Trial	
**Acronym**	EU-SolidAct
**Registry Number**	NCT04891133, 2021-000541-41
**Master protocol**	https://eu-response.eu/eu-solidact/#1625141473942-0445f558-1680
**Results**	Trøseid M., Arribas J. R. et al. Efficacy and safety of baricitinib in hospitalized adults with severe or critical COVID-19 (Bari-SolidAct): a randomised, double-blind, placebo-controlled phase 3 trial
A platform trial in practice: adding a new experimental research arm to the ongoing confirmatory FLAIR trial in chronic lymphocytic leukaemia	
**Acronym**	FLAIR Trial
**Registry Number**	2013-001944-76, ISRCTN01844152
**Master protocol**	Howard R. D., Hockaday A. et al. A platform trial in practice: adding a new experimental research arm to the ongoing confirmatory FLAIR trial in chronic lymphocytic leukaemia<br>Collet L., Howard R. D. et al. Assessment of ibrutinib plus rituximab in front-line CLL (FLAIR trial): study protocol for a phase III randomised controlled trial
**Results**	Hillmen P., Pitchford A. et al. Ibrutinib Plus Rituximab Is Superior to FCR in Previously Untreated CLL: Results of the Phase III NCRI FLAIR Trial<br>Hillmen et al., Ibrutinib and rituximab versus fludarabine, cyclophosphamide, and rituximab for patients with previously untreated chronic lymphocytic leukaemia (FLAIR): interim analysis of a multicentre, open-label, randomised, phase 3 trial, The Lancet Oncology<br>Hillmen et al., S145: THE COMBINATION OF IBRUTINIB PLUS VENETOCLAX RESULTS IN A HIGH RATE OF MRD NEGATIVITY IN PREVIOUSLY UNTREATED CLL: THE RESULTS OF THE PLANNED INTERIM ANALYSIS OF THE PHASE III NCRI FLAIR TRIAL
FOCUS4: Molecular selection of therapy in colorectal cancer: a molecularly stratified randomised controlled trial programme	
**Acronym**	FOCUS4
**Registry Number**	2012-005111-12, ISRCTN90061546
**Master protocol**	Kaplan R. The FOCUS4 design for biomarker stratified trials<br>Schmoll J. H. FOCUS4: a new trial design for evaluation of targeted drugs in colorectal cancer?
**Results**	Seligmann F. J., Fisher J. D. et al. Inhibition of WEE1 Is Effective in *TP53*- and *RAS*-Mutant Metastatic Colorectal Cancer: A Randomized Trial (FOCUS4-C) Comparing Adavosertib (AZD1775) With Active Monitoring<br>Adams R., Brown E. et al. Inhibition of EGFR, HER2, and HER3 signalling in patients with colorectal cancer wild-type for *BRAF, PIK3CA, KRAS*, and *NRAS* (FOCUS4-D): a phase 2–3 randomised trial<br>Kasi M. P. Treatment Holidays for Patients With Metastatic Colorectal Cancer
FRACTION (Fast Real-time Assessment of Combination Therapies in Immuno-ONcology)-gastric cancer (GC): A randomized, open-label, adaptive, phase 2 study of nivolumab in combination with other immuno-oncology (IO) agents in patients with advanced GC	
**Acronym**	FRACTION_GC
**Registry Number**	NCT02935634, 2016-002807-24
**Master protocol**	Simonsen L. K., Fracasso M. P. et al. The Fast Real-time Assessment of Combination Therapies in Immuno-ONcology (FRACTION) program: innovative, high-throughput clinical screening of immunotherapies<br>Aanur P., Gutierrez M. et al. FRACTION (Fast Real-time Assessment of Combination Therapies in Immuno-Oncology)-gastric cancer (GC): A randomized, open-label, adaptive, phase 2 study of nivolumab in combination with other immuno-oncology (IO) agents in patients with advanced GC.
**Results**	Not available
FRACTION-RCC: a randomized, open-label, adaptive, phase 2 study of nivolumab in combination with other immuno-oncology agents in patients with advanced RCC	
**Acronym**	FRACTION_RCC
**Registry Number**	NCT02996110, 2016-003082-26
**Master protocol**	Simonson L. K., Fracasso M. P. et al. The Fast Real-time Assessment of Combination Therapies in Immuno-ONcology (FRACTION) program: innovative, high-throughput clinical screening of immunotherapies
**Results**	Simonson L. K., Fracasso M. P. et al. The Fast Real-time Assessment of Combination Therapies in Immuno-ONcology (FRACTION) program: innovative, high-throughput clinical screening of immunotherapies
A phase 2 trial, fast real-time assessment of combination therapies in immuno-oncology, in patients with advanced non-small cell lung cancer (FRACTION-lung)	
**Acronym**	FRACTION_LUNG
**Registry Number**	NCT02750514
**Master protocol**	Simonsen L. K., Fracasso M. P. et al. The Fast Real-time Assessment of Combination Therapies in Immuno-ONcology (FRACTION) program: innovative, high-throughput clinical screening of immunotherapies

	Fracasso M.P., Freeman J.D. et al. A phase 2, fast real-time assessment of combination therapies in immuno-oncology trial in patients with advanced non-small cell lung cancer (FRACTION-lung)
**Results**	https://www.clincosm.com/trial/advanced-cancer-international-nivolumab-dasatinib-relatlimab
Adaptive global innovative learning environment for glioblastoma: GBM AGILE	
**Acronym**	GBM AGILE
**Registry Number**	NCT03970447
**Master protocol**	Alexander M. B., Ba S. et al. Adaptive Global Innovative Learning Environment for Glioblastoma: GBM AGILE<br>Buxton B. M., Alexander M. B. et al. GBM AGILE: A global, phase II/III adaptive platform trial to evaluate multiple regimens in newly diagnosed and recurrent glioblastoma.<br>Wen Y. P., Mellinghoff K. I. et al. GBM AGILE: A global, phase 2/3 adaptive platform trial to evaluate multiple regimens in newly diagnosed and recurrent glioblastoma.
**Results**	Not available
A platform trial for ALS: Innovative trial design to drive ALS science and accelerate the path to effective treatments	
**Acronym**	HEALY
**Registry Number**	NCT04297683, NCT04615923, NCT04414345, NCT04436497, NCT04436510, NCT05136885
**Master protocol**	Paganoni S., Berry D. J. et al. Adaptive Platform Trials to Transform Amyotrophic Lateral Sclerosis Therapy Development
**Results**	Not available
Randomized, Double Blind, Placebo-controlled Phase 2b Study to Evaluate Efficacy, Pharmacokinetics, and Safety of 48-week Study Intervention With JNJ 73763989+JNJ 56136379+Nucleos(t)Ide Analog (NA) Registry Numberimen Compared to NA Alone in e Antigen Negative Virologically Suppressed Participants With Chronic Hepatitis B Virus Infection	
**Acronym**	HepB Wings Platform
**Registry Number**	NCT04129554, 2019-002674-31
**Master protocol**	Not available
**Results**	Agarwal K., Buti M. et al. Efficacy and Safety of Finite 48-week Treatment With the siRNA JNJ-3989 and the Capsid Assembly Modulator JNJ-6379 in HBeAg Negative Virologically Suppressed Chronic Hepatitis B Patients: Results from the REEF-2 Study
Phase III INHIBIT Platform: Prevention Trial, Eloctate vs Emicizumab to Prevent Inhibitors; Eradication Trial: Eloctate Immune Tolerance (ITI) Plus Emicizumab vs vs Eloctate ITI Alone to Eradicate Inhibitors in Severe Hemophilia A	
**Acronym**	INHIBIT
**Registry Number**	NCT02196207
**Master protocol**	Bertolet M., Brooks M. M. et al. The design of a Bayesian platform trial to prevent and eradicate inhibitors in patients with hemophilia
**Results**	Not available
Individualized screening trial of innovative glioblastoma therapy (INSIGhT): A Bayesian adaptive platform trial to develop precision medicines for patients with glioblastoma	
**Acronym**	INSIGhT
**Registry Number**	NCT02977780
**Master protocol**	Alexander M. B., Trippa L. et al. Individualized Screening Trial of Innovative Glioblastoma Therapy (INSIGhT): A Bayesian Adaptive Platform Trial to Develop Precision Medicines for Patients With Glioblastoma<br>Alexander M. B., Trippa L. et al. Individualized screening trial of innovative glioblastoma therapy (INSIGhT).
**Results**	Lee Q. E., Trippa L. et al. Preliminary results of the abemaciclib arm in the Individualized Screening Trial of Innovative Glioblastoma Therapy (INSIGhT): A phase II platform trial using Bayesian adaptive randomization.<br>Rahman R., Trippa L. et al. Evaluating the benefit of adaptive randomization in the CC-115 arm of the Individualized Screening Trial of Innovative Glioblastoma Therapy (INSIGhT): A phase II randomized Bayesian adaptive platform trial in newly diagnosed MGMT unmethylated glioblastoma.
A prescription for new trial designs for drug development focused on the neoadjuvant setting: Save lives, resources, and time	
**Acronym**	I-SPY
**Registry Number**	NCT01042379, NCT00043017
**Master protocol**	Barker D. A., Sigman C. C. et al. I-SPY 2: an adaptive breast cancer trial design in the setting of neoadjuvant chemotherapy<br>Park W. J., Liu C. M. et al. Adaptive Randomization of Neratinib in Early Breast Cancer<br>Das S., Lo W. A. Adaptive Randomization of Neratinib in Early Breast Cancer
**Results**	Park J. W., Liu M. C., Yee D., et al. Adaptive Randomization of Neratinib in Early Breast Cancer. N Engl J Med 375: 11-22, 2016.<br>Park J. W., Liu M. C., Yee D., et al. Abstract CT227: Neratinib plus standard neoadjuvant therapy for high-risk breast cancer: Efficacy results from the I-SPY 2 TRIAL<br>Rugo H. S., Olopade, O. I., DeMichele A. et al. Adaptive randomization of veliparib-carboplatin treatment in breast cancer. New Engl J Med 375: 23-34, 2016.<br>Rugo H. S., Olopade, O. I., DeMichele A. et al. Abstract S5-02: Veliparib/carboplatin plus standard neoadjuvant therapy for high-risk breast cancer: First efficacy results from the I-SPY 2 TRIAL<br>Albain K. S., Leyland-Jones B., Symmans F. Abstract P1-14-03: The evaluation of trebananib plus standard neoadjuvant therapy in high-risk breast cancer: Results from the I-SPY 2 TRIAL

© 2024 Griessbach A et al. *JAMA Network Open.*

	Douglas Y., Isaacs C. et al. Ganitumab and metformin plus standard neoadjuvant therapy in stage 2/3 breast cancer<br>Yee D., Paoloni M. et al. Abstract P6-11-04: The evaluation of ganitumab/metformin plus standard neoadjuvant therapy in high-risk breast cancer: Results from the I-SPY 2 trial<br>Chien J. A., Tripathy D. et al. MK-2206 and Standard Neoadjuvant Chemotherapy Improves Response in Patients with Human Epidermal Growth Factor Receptor 2-Positive and/or Hormone Receptor-Negative Breast Cancers in the I-SPY 2 Trial<br>Buxton M., DeMichele M. A. et al. Abstract CT106: Efficacy of pertuzumab/trastuzumab/paclitaxel over standard trastuzumab/paclitaxel therapy for HER2+ breast cancer: Results from the neoadjuvant I-SPY 2 TRIAL<br>Clark S. A., Yau C. et al. Neoadjuvant T-DM1/pertuzumab and paclitaxel/trastuzumab/pertuzumab for $HER2^+$ breast cancer in the adaptively randomized I-SPY2 trial<br>Clark S. A., Yau C. et al. Neoadjuvant T-DM1/pertuzumab and paclitaxel/trastuzumab/pertuzumab for $HER2^+$ breast cancer in the adaptively randomized I-SPY2 trial<br>Forero A., Yee D. et al. Abstract P6-11-02: Efficacy of Hsp90 inhibitor ganetespib plus standard neoadjuvant therapy in high-risk breast cancer: Results from the I-SPY 2 trial<br>Nanda R., Minetta C. Liu et al. Effect of Pembrolizumab Plus Neoadjuvant Chemotherapy on Pathologic Complete Response in Women With Early-Stage Breast CancerAn Analysis of the Ongoing Phase 2 Adaptively Randomized I-SPY2 Trial<br>Nanda R., Minetta C. L. et al. Pembrolizumab plus standard neoadjuvant therapy for high-risk breast cancer (BC): Results from I-SPY 2.<br>Schwab R., Clark S. A. et al. Abstract CT136: Evaluation of talazoparib in combination with irinotecan in early stage, high-risk HER2 negative breast cancer: Results from the I-SPY 2 TRIAL<br>Helsten L. T., Shelly S. Lo. Abstract P3-11-02: Evaluation of patritumab/paclitaxel/trastuzumab over standard paclitaxel/trastuzumab in early stage, high-risk HER2 positive breast cancer: Results from the neoadjuvant I-SPY 2 trial<br>Liu M. C., Robinson P. A. et al. Evaluation of a pembrolizumab-8 cycle neoadjuvant regimen without AC for high-risk early-stage HER2-negative breast cancer: Results from the I-SPY 2 TRIAL<br>DiGiorgio K. New Data From Phase 2 I-SPY 2 TRIAL Shows Improved Outcomes with Combination of Merck's KEYTRUDA® (pembrolizumab) Plus Standard Neoadjuvant Therapy in Patients with High-Risk Breast Cancer<br>Beckwith H., Schwab R. et al. Abstract PD1-10: Evaluation of SGN-LIV1a followed by AC in high-risk HER2 negative stage II/III breast cancer: Results from the I-SPY 2 TRIAL<br>Pusztai L., Hyo S. et al. Abstract CT011: Evaluation of durvalumab in combination with olaparib and paclitaxel in high-risk HER2 negative stage II/III breast cancer: Results from the I-SPY 2 TRIAL<br>Chien J. A., Soliman H. H. et al. Evaluation of intra-tumoral (IT) SD-101 and pembrolizumab (Pb) in combination with paclitaxel (P) followed by AC in high-risk HER2-negative (HER2-) stage II/III breast cancer: Results from the I-SPY 2 trial.
I-SPY COVID TRIAL: An Adaptive Platform Trial to Reduce Mortality and Ventilator Requirements for Critically Ill Patients	
**Acronym**	I-SPY COVID
**Registry Number**	NCT04488081
**Master protocol**	Files C. D., Matthay A. M. et al. I-SPY COVID adaptive platform trial for COVID-19 acute respiratory failure: rationale, design and operations
**Results**	DiGiorgio K. Quantum Leap Healthcare Collaborative Concludes Cenicriviroc Not Likely to Reduce Time to Recovery or Mortality in Critically Ill Patients in I-SPY COVID Trial<br>DiGiorgio K. I-SPY COVID Trial Sponsored by Quantum Leap Healthcare Collaborative Suggests Addition of Icatibant is Unlikely to Impact Critically Ill COVID-19 Patients<br>DiGiorgio K. Quantum Leap Healthcare Collaborative Discontinues Testing of Aerpio Pharmaceutical's Razuprotafib in I-SPY COVID Trial<br>DiGiorgio K. Quantum Leap Healthcare Collaborative Concludes Apremilast Not Likely to Reduce Time to Recovery or Mortality in Critically Ill Patients in I-SPY COVID Trial<br>DiGiorgio K. No Clinical Benefit Suggested by Addition of Pulmozyme® to Treatment of Critically Ill Patients With COVID-19 in I-SPY COVID Trial<br>DiGiorgio K. I-SPY COVID Trial Suggests No Clinical Benefit from Addition of IC14 to Treatment of Critically Ill Patients with COVID-19<br>DiGiorgio K. Quantum Leap Healthcare Collaborative Concludes Famotidine Celebrex combination Does Not Reduce Time to Recovery or Mortality in Critically Ill Patients in the I-SPY COVID Trial<br>DiGiorgio K. Quantum Leap Healthcare Collaborative Announces Termination of the Cyclosporine Treatment Arm for Critically Ill COVID-19 Patients in the I-SPY COVID Trial<br>DiGiorgo K. I-SPY COVID Trial Sponsored by Quantum Leap Healthcare Collaborative Reports Assessment of Narsoplimab for Treatment of Critically Ill Patients With COVID-19
A Phase 1/2 Open-Label Rolling-Arm Umbrella Platform Design of Investigational Agents With or Without Pembrolizumab or Pembrolizumab Alone in Participants with Melanoma (KEYNOTE-U02)	
**Acronym**	KEYNOTE-U02/KEYMAKER
**Registry Number**	NCT04305041, NCT04305054, NCT04303169, NCT04700072, 2019-003956-35, 2019-003977-24, 2019-003978-22,2020-003742-36

© 2024 Griessbach A et al. *JAMA Network Open.*

**Master protocol**	Dummer R., Long V. G. et al. 426 MK-3475-U02: Phase 1/2 study of investigational agents with or without pembrolizumab versus pembrolizumab monotherapy in melanoma<br>Dummer R., Long V. G. et al. 1092TiP Investigational (Inv) agents with or without pembrolizumab (pembro) or pembro alone in melanoma (mel): KEYMAKER-U02
**Results**	Not available
A Randomized Phase II/III Trial of "Novel Therapeutics" Versus Azacitidine in Newly Diagnosed Patients With Acute Myeloid Leukemia (AML) or High-Risk Myelodysplastic Syndrome (MDS), Age 60 or Older LEAP: Less-Intense AML Platform Trial	
**Acronym**	LEAP S1612
**Registry Number**	NCT03092674
**Master protocol**	Walter B. R., Michaelis C. L. et al. Intergroup LEAP trial (S1612): A randomized phase 2/3 platform trial to test novel therapeutics in medically less fit older adults with acute myeloid leukemia
**Results**	Hay E. A., Assouline S. et al. Accrual Barriers and Detection of Early Toxicity Signal in Older Less-Fit Patients Treated with Azacitidine and Nivolumab for Newly Diagnosed Acute Myeloid Leukemia (AML) or High-Risk Myelodysplastic Syndrome (MDS) in the SWOG 1612 Platform Randomized Phase II/III Clinical Trial
Phase 2 Study of Talazoparib in Patients With Homologous Recombination Repair	
**Acronym**	LUNG-MAP
**Registry Number**	NCT02154490, NCT03851445, NCT03845296, NCT04267913, NCT04625647, NCT04268550, NCT03574753, NCT02785939, NCT04173507, NCT03971474, NCT03377556, NCT02965378, NCT02785913, NCT02766335, NCT02785952, NCT02926638, NCT03373760, NCT05096663
**Master protocol**	Ferrarotto R., Redman W. M. et al. Lung-MAP—framework, overview, and design principles<br>Steuer E. C., Papadimitrakopoulou V. et al. Innovative Clinical Trials: The LUNG-MAP Study<br>Redman W. M., Minichiello K. et al. Biomarker-driven therapies for previously treated squamous non-small-cell lung cancer (Lung-MAP SWOG S1400): a biomarker-driven master protocol
**Results**	Papadimitrakopoulou V., Redman W. M. et al. A phase II study of durvalumab (MEDI4736) for previously treated patients with stage IV squamous NSCLC (SqNSCLC): Lung-MAP Sub-study SWOG S1400A<br>Borghaei H., Redman W. M. et al. SWOG S1400A (NCT02154490): A Phase II Study of Durvalumab for Patients With Previously Treated Stage IV or Recurrent Squamous Cell Lung Cancer (Lung-MAP Sub-study)<br>Wade L. J., Langer J. C. et al. A phase II study of GDC-0032 (taselisib) for previously treated PI3K positive patients with stage IV squamous cell lung cancer (SqNSCLC): LUNG-MAP sub-study SWOG S1400B.<br>Langer J. C., Redman W. M. et al. SWOG S1400B (NCT02785913), a Phase II Study of GDC-0032 (Taselisib) for Previously Treated PI3K-Positive Patients with Stage IV Squamous Cell Lung Cancer (Lung-MAP Sub-Study)<br>Edelman J. M., Redman W. M. et al. SWOG S1400C (NCT02154490)—A Phase II Study of Palbociclib for Previously Treated Cell Cycle Gene Alteration–Positive Patients with Stage IV Squamous Cell Lung Cancer (Lung-MAP Substudy)<br>Edelman J. M., Redman W. M. et al. A phase II study of palbociclib (P) for previously treated cell cycle gene alteration positive patients (pts) with stage IV squamous cell lung cancer (SCC): Lung-MAP sub-study SWOG S1400C.<br>Aggarwal C. Redman W. M. et al. SWOG S1400D (NCT02965378), a Phase II Study of the Fibroblast Growth Factor Receptor Inhibitor AZD4547 in Previously Treated Patients With Fibroblast Growth Factor Pathway–Activated Stage IV Squamous Cell Lung Cancer (Lung-MAP Substudy)<br>Aggarwal C., Redman W. M., Phase II study of the FGFR inhibitor AZD4547 in previously treated patients with FGF pathway-activated stage IV squamous cell lung cancer (SqNSCLC): LUNG-MAP sub-study SWOG S1400D.<br>Papadimitrakopoulou V. et al. Lung-MAP: Rilotumumab and Erlotinib Hydrochloride or Erlotinib Hydrochloride Alone as Second-Line Therapy in Treating Patients With Recurrent Stage IV Squamous Cell Lung Cancer and Positive Biomarker Matches, ClinicalTrials.gov<br>Bazhenova L., Redman W. M. et al. A phase III randomized study of nivolumab plus ipilimumab versus nivolumab for previously treated patients with stage IV squamous cell lung cancer and no matching biomarker (Lung-MAP Sub-Study S1400I, NCT02785952).<br>Reckamp L. K., Redman W. M. et al. Phase II randomized study of ramucirumab plus pembrolizumab versus standard of care for advanced non-small cell lung cancer previously treated with a checkpoint inhibitor: Toxicity update (Lung-MAP non-matched sub-study S1800A).<br>Reckamp L. K., Redman W. M. et al., Phase II Randomized Study of Ramucirumab and Pembrolizumab Versus Standard of Care in Advanced Non–Small-Cell Lung Cancer Previously Treated With Immunotherapy—Lung-MAP S1800A
MORPHEUS: A Study Evaluating the Efficacy and Safety of Multiple Immunotherapy-Based Treatment Combinations in Patients With Advanced Liver Cancers	
**Acronym**	MORPHEUS-Liver
**Registry Number**	NCT04524871
**Master protocol**	Not available
**Results**	Not available

© 2024 Griessbach A et al. *JAMA Network Open.*

Industry Alliance Platform Trial to Assess the Efficacy and Safety of Multiple Candidate Agents for the Treatment of COVID-19 in Hospitalized Patients	
**Acronym**	COMMUNITY
**Registry Number**	NCT04590586, 2020-002594-10
**Master protocol**	Not available
**Results**	Not available
Efficacy of Novel Agents for Treatment of SARS-CoV-2 Infection Among High-Risk Outpatient Adults: An Adaptive Randomized Platform Trial	
**Acronym**	Not available
**Registry Number**	NCT04354428
**Master protocol**	Not available
**Results**	Johnston C., Brown R. E. et al. Hydroxychloroquine with or without azithromycin for treatment of early SARS-CoV-2 infection among high-risk outpatient adults: A randomized clinical trial
A Randomized, Subject and Investigator Blinded, Placebo-controlled and Multi-center Platform Study, to Assess Efficacy and Safety of Different Investigational Drugs in Patients With Moderate to Severe Hidradenitis Suppurativa	
**Acronym**	Not available
**Registry Number**	NCT03827798, 2018-002757-30
**Master protocol**	Not available
**Results**	Not available
A Phase 2 Open-label, Multicenter, Randomized, Multidrug Platform Study of Neoadjuvant Durvalumab Alone or in Combination with Novel Agents in Subjects with Resectable, Early-stage (I [> 2 cm] to IIIA) Non-small Cell Lung Cancer (NeoCOAST)	
**Acronym**	NeoCOAST
**Registry Number**	NCT03794544, 2018-002932-26
**Master protocol**	R. Garcia Campelo et al, P2.04-28 NeoCOAST: Neoadjuvant Durvalumab Alone or with Novel Agents for Resectable, Early-Stage (I–IIIA) Non-Small Cell Lung CancerClinicalTrial.gov A Phase 2 Open-label, Multicenter, Randomized, Multidrug Platform Study of Neoadjuvant Durvalumab Alone or in Combination with Novel Agents in Subjects with Resectable, Early-stage (I [> 2 cm] to IIIA) Non-small Cell Lung Cancer (NeoCOAST)
**Results**	Spiecer et al. , 929MO Platform study of neoadjuvant durvalumab (D) alone or combined with novel agents in patients (pts) with resectable, early-stage non-small cell lung cancer (NSCLC): Pharmacodynamic correlates and circulating tumor DNA (ctDNA) dynamics in the NeoCOAST study
A Phase II Clinical Trial Platform of Sensitization Utilizing Total Neoadjuvant Therapy (TNT) in Rectal Cancer	
**Acronym**	NRG-GI002
**Registry Number**	NCT02921256
**Master protocol**	George J. T., Yothers G. et al. NRG-GI002: A phase II clinical trial platform for total neoadjuvant therapy (TNT) in rectal cancer.<br>Thomas J. George et al, NRG-GI002: A phase II clinical trial platform for total neoadjuvant therapy (TNT) in rectal cancer.
**Results**	Rahma E. O., Yothers G. et al. Use of Total Neoadjuvant Therapy for Locally Advanced Rectal Cancer: Initial Results From the Pembrolizumab Arm of a Phase 2 Randomized Clinical Trial<br>Rahma E. O., Yothers G. et al. NRG-GI002: A phase II clinical trial platform using total neoadjuvant therapy (TNT) in locally advanced rectal cancer (LARC)—Pembrolizumab experimental arm (EA) primary results.<br>George J. T., Yothers G. et al. NRG-GI002: A phase II clinical trial platform using total neoadjuvant therapy (TNT) in locally advanced rectal cancer (LARC)—First experimental arm (EA) initial results.
A Phase II, Randomized, Open-label Platform Trial Utilizing a Master Protocol to Study Novel Registry Numberimens Versus Standard of Care Treatment in NSCLC Participants	
**Acronym**	NSCL Trial
**Registry Number**	NCT03739710, 2018-001316-29
**Master protocol**	Spigel D., Garassino M. et al. P1.01-110 Novel Regimens Versus Standard-of-Care in NSCLC: A Phase II, Randomized, Open-Label, Platform Trial Using a Master Protocol<br>Spigel R.D., Lopez G.P. et al. 1189TiP Phase II study of anti-TIGIT GSK4428859A (GSK'859A)/EOS-448 + anti-CD96 GSK6097608 (GSK'608) + anti-PD-1 dostarlimab in non-small cell lung cancer (NSCLC)
**Results**	Not available
A Multicenter, Multi-Outbreak, Randomized, Controlled Safety and Efficacy Study of Investigational Therapeutics for the Treatment of Patients With Ebola Virus Disease	
**Acronym**	PALM
**Registry Number**	NCT03719586
**Master protocol**	Not available
**Results**	Mulangu S., Dodd E. L. et al. A Randomized, Controlled Trial of Ebola Virus Disease Therapeutics<br>Mulangu S. Abstract: 843. The PALM Consortium: A Multicenter, Multioutbreak Randomized Controlled Trial of Ebola Virus Disease Therapeutics
Precision Promise Platform Trial for Metastatic Pancreatic Cancer	
**Acronym**	PanCan Precision Promise

© 2024 Griessbach A et al. *JAMA Network Open.*

**Registry Number**	NCT04229004
**Master protocol**	Picozzi J. V., Duliege Anne-Marie et al. Abstract PO-050: Precision Promise (PrP): An adaptive, multi-arm registration trial in metastatic pancreatic ductal adenocarcinoma (PDAC)<br>Noel S. M., Wong S. et al. Oral SM-88 plus MPS, an effective yet less toxic treatment option in second-line advanced pancreatic cancer? Final phase II/III study results.
**Results**	Not available
Prostate Adenocarcinoma: TransCutaneous Hormones, PR09 (PATCH): A randomized controlled trial of transdermal estrogen patches versus luteinising hormone releasing hormone agonists in locally advanced and metastatic prostate cancer	
**Acronym**	PATCH
**Registry Number**	NCT00303784, EUDRACT-2005-001030-33, ISRCTN70406718
**Master protocol**	Langley E. R., Duong T. et al. Prostate Adenocarcinoma: TransCutaneous Hormones, PR09 (PATCH): A randomized controlled trial of transdermal estrogen patches versus luteinising hormone releasing hormone agonists in locally advanced and metastatic prostate cancer.<br>Langley E. R., Kynaston G. H. et al. A Randomised Comparison Evaluating Changes in Bone Mineral Density in Advanced Prostate Cancer: Luteinising Hormone-releasing Hormone Agonists Versus Transdermal Oestradiol<br>Gilbert C. D., Duong T. et al. Quality-of-life outcomes from the Prostate Adenocarcinoma: TransCutaneous Hormones (PATCH) trial evaluating luteinising hormone-releasing hormone agonists versus transdermal oestradiol for androgen suppression in advanced prostate cancer
**Results**	Langley E. R.,et al. Transdermal oestradiol for androgen suppression in prostate cancer: long-term cardiovascular outcomes from the randomised Prostate Adenocarcinoma Transcutaneous Hormone (PATCH) trial programme<br>Langley E. R., Duong T. et al. Bone density in men receiving androgen deprivation therapy for prostate cancer: A randomized comparison between transdermal estrogen and luteinising hormone-releasing hormone agonists.<br>Irmak B., Bölükbas N. et al. The life quality of patients who receiving chemotherapy treatment after mastectomy and stoma surgery
PIONEER-Panc: Phase II Investigations of New and Emerging Therapies for Pancreatic Cancer	
**Acronym**	PIONEER-Panc
**Registry Number**	NCT04481204
**Master protocol**	Douglas E. J., Liu S. et al. PIONEER-Panc: a platform trial for phase II randomized investigations of new and emerging therapies for localized pancreatic cancer
**Results**	Not available
A Trial To Evaluate The Efficacy And Safety Of Multiple Combination Therapies In Participants With Chronic Hepatitis B (Piranga)	
**Acronym**	Piranga
**Registry Number**	NCT04225715, 2019-002086-35
**Master protocol**	Not available
**Results**	Not available
PLATFORM: Planning treatment of oesophago-gastric (OG) cancer randomised maintenance therapy trial	
**Acronym**	PLATFORM
**Registry Number**	NCT02678182, 2014-002169-30
**Master protocol**	Cafferkey C., Chau I. et al. PLATFORM: Planning treatment of oesophago-gastric (OG) cancer—A randomised maintenance therapy trial.
**Results**	Fong C., Patel B. et al. Maintenance durvalumab after first-line platinum-based chemotherapy in advanced oesophago-gastric (OG) adenocarcinoma: Results from the PLATFORM trial.<br>Chunningham D., Fong K. Y. C. et al. Evaluating maintenance therapies in advanced oesophago-gastric adenocarcinoma (OGA): Interim analysis and biomarker results from the PLATFORM study.
A Randomized, Open-label, Phase II Open Platform Study Evaluating the Efficacy and Safety of Novel Spartalizumab (PDR001) Combinations in Previously Treated Unresectable or Metastatic Melanoma	
**Acronym**	PLATforM
**Registry Number**	NCT03484923, 2018-000610-38
**Master protocol**	Weber et al, A randomized, open-label, phase II open platform study evaluating the efficacy and safety of novel spartalizumab (PDR001) combinations in previously treated unresectable or metastatic melanoma (PLATForM)<br>Caroline Robert et al., Abstract CT120: A randomized, open-label, open-platform, Phase II study evaluating the efficacy and safety of novel spartalizumab (PDR001) combinations in previously treated unresectable or metastatic melanoma (PLATforM)
**Results**	Robert C., Schadendorf D. et al. 1084P PLATForM: Descriptive analysis from a randomised, phase II study of novel spartalizumab combinations in previously treated unresectable/metastatic melanoma
PrecISE (Precision Interventions for Severe and/or Exacerbation-Prone Asthma) Network Study	
**Acronym**	PRECISE
**Registry Number**	NCT04129931
**Master protocol**	Israel E., Denlinger C. L. et al. PrecISE: Precision Medicine in Severe Asthma: An adaptive platform trial with biomarker ascertainment

© 2024 Griessbach A et al. *JAMA Network Open.*

	Ivanova A., Israel E. et al. The precision interventions for severe and/or exacerbation-prone asthma (PrecISE) adaptive platform trial: statistical considerations
**Results**	Not available
A Multicenter Randomized Safety and Efficacy Study of Putative Investigational Therapeutics in the Treatment of Patients With Known Ebola Infection	
**Acronym**	PREVAILII
**Registry Number**	NCT02363322
**Master protocol**	Dodd E. L., Proschan A. M. et al. Design of a Randomized Controlled Trial for Ebola Virus Disease Medical Countermeasures: PREVAIL II, the Ebola MCM Study
**Results**	Massachusetts Medical Society, A Randomized, Controlled Trial of ZMapp for Ebola Virus Infection, New England Journal of Medicine
Platform Randomised trial of INterventions against COVID-19 In older people (PRINCIPLE)	
**Acronym**	PRINCIPLE
**Registry Number**	2020-001209-22, ISRCTN86534580
**Master protocol**	Hayward G., Butler C. C. et al. Platform Randomised trial of INterventions against COVID-19 In older peoPLE (PRINCIPLE): protocol for a randomised, controlled, open-label, adaptive platform, trial of community treatment of COVID-19 syndromic illness in people at higher risk
**Results**	Butler C. C., Dorward J. et al. Azithromycin for community treatment of suspected COVID-19 in people at increased risk of an adverse clinical course in the UK (PRINCIPLE): a randomised, controlled, open-label, adaptive platform trial<br>Butler C. C., Dorward J. et al. Azithromycin for community treatment of suspected COVID-19 in people at increased risk of an adverse clinical course in the UK (PRINCIPLE): a randomised, controlled, open-label, adaptive platform trial<br>Butler C. C., Yu L. et al. Doxycycline for community treatment of suspected COVID-19 in people at high risk of adverse outcomes in the UK (PRINCIPLE): a randomised, controlled, open-label, adaptive platform trial<br>Yu L., Bafadhel M. et al. Inhaled budesonide for COVID-19 in people at high risk of complications in the community in the UK (PRINCIPLE): a randomised, controlled, open-label, adaptive platform trial<br>Dorward J., Yu L. et al. Colchicine for COVID-19 in adults in the community (PRINCIPLE): a randomised, controlled, adaptive platform trial
The ProBio trial: molecular biomarkers for advancing personalized treatment decision in patients with metastatic castration-resistant prostate cancer	
**Acronym**	ProBio
**Registry Number**	NCT03903835, 2018-002350-78
**Master protocol**	Crippa A., De Laere B. et al. The ProBio trial: molecular biomarkers for advancing personalized treatment decision in patients with metastatic castration-resistant prostate cancer<br>Lindberg et al., ProBio: An outcome-adaptive, multi-arm, open-label, multiple assignment randomised controlled biomarker-driven trial in patients with metastatic castration-resistant prostate cancer
**Results**	Not available
An International Investigator-led Phase III Multi Arm Multi Stage Multi-centre Randomised Controlled Platform Trial of Adjuvant Therapy in Patients With Resected Primary Renal Cell Carcinoma (RCC) at High or Intermediate Risk of Relapse	
**Acronym**	RAMPART
**Registry Number**	NCT03288532, 2017-002329-39, ISRCTN53348826
**Master protocol**	Oza et al., RAMPART: A phase III multi-arm multi-stage trial of adjuvant checkpoint inhibitors in patients with resected primary renal cell carcinoma (RCC) at high or intermediate risk of relapse<br>Meade et al., RAMPART: A model for a regulatory-ready academic-led phase III trial in the adjuvant renal cell carcinoma setting<br><br>Larkin J. Renal Adjuvant MultiPle Arm Randomised Trial (RAMPART): An international investigator-led phase III multi-arm multistage multicentre randomized controlled platform trial of adjuvant therapy in patients with resected primary renal cell carcinoma (RCC) at high or intermediate risk of relapse
**Results**	Not available
Randomised Evaluation of COVID-19 Therapy (RECOVERY)	
**Acronym**	Randomised Evaluation of COVID-19 Therapy (RECOVERY)
**Registry Number**	NCT04381936, 2020-001113-21, ISRCTN50189673
**Master protocol**	see protocol on website
**Results**	Dexamethasone in Hospitalized Patients with Covid-19, The RECOVERY Collaborative Group*<br><br>Press release: https://www.recoverytrial.net/files/recovery_dexamethasone_statement_160620_v2final.pdf<br><br>Preprint: Horby et al., Effect of Hydroxychloroquine in Hospitalized Patients with COVID-19: Preliminary results from a multi-centre, randomized, controlled trial<br><br>Press release: https://www.recoverytrial.net/files/hcq-recovery-statement-050620-final-002.pdf

© 2024 Griessbach A et al. *JAMA Network Open.*

	Lopinavir–ritonavir in patients admitted to hospital with COVID-19 (RECOVERY): a randomised, controlled, open-label, platform trial, RECOVERY Collaborative Group<br><br>Press release: https://www.recoverytrial.net/files/lopinavir-ritonavir-recovery-statement-29062020_final.pdf<br>Publiucation: Azithromycin in patients admitted to hospital with COVID-19 (RECOVERY): a randomised, controlled, open-label, platform trial, RECOVERY Collaborative Group<br><br>Preprint: Horby et al., Azithromycin in Hospitalised Patients with COVID-19 (RECOVERY): a randomised, controlled, open-label, platform trial<br><br>Press release: https://www.recoverytrial.net/files/azithromycin-recovery-statement-141220_final.pdf<br><br>Publication: Tocilizumab in patients admitted to hospital with COVID-19 (RECOVERY): a randomised, controlled, open-label, platform trial, RECOVERY Collaborative Group<br><br>Wood C. RECOVERY trial finds no benefit from azithromycin in patients hospitalised with COVID-19<br>Publication: Convalescent plasma in patients admitted to hospital withCOVID-19 (RECOVERY): a randomised controlled, open-label, platform trial, RECOVERY Collaborative Group*<br><br>Preprint: Convalescent plasma in patients admitted to hospital with COVID-19 (RECOVERY): a randomised, controlled, open-label, platform trial, The RECOVERY Collaborative Group<br><br>Press release: https://www.recoverytrial.net/files/recovery_publicstatement_cp_2021-01-15_final.pdf<br>Abani O., Abbas A. et al. Casirivimab and imdevimab in patients admitted to hospital with COVID-19 (RECOVERY): a randomised, controlled, open-label, platform trial<br>Horby W. P., Mafham M. et al. Casirivimab and imdevimab in patients admitted to hospital with COVID-19 (RECOVERY): a randomised, controlled, open-label, platform trial<br>Wood C. et al. RECOVERY trial finds Regeneron's monoclonal antibody combination reduces deaths for hospitalised COVID-19 patients who have not mounted their own immune response<br>Abani O., Abbas A. et al. Aspirin in patients admitted to hospital with COVID-19 (RECOVERY): a randomised, controlled, open-label, platform trial<br>Horby W. P., Pessoa-Amorim G. et al. Aspirin in patients admitted to hospital with COVID-19 (RECOVERY): a randomised, controlled, open-label, platform trial<br>Wood C. RECOVERY trial finds aspirin does not improve survival for patients hospitalised with COVID-19<br>Horby W. P., Campbell M. et al. Colchicine in patients admitted to hospital with COVID-19 (RECOVERY): a randomised, controlled, open-label, platform trial<br>Horby W. P., Campbell M. et al. Colchicine in patients admitted to hospital with COVID-19 (RECOVERY): a randomised, controlled, open-label, platform trial<br>Wood C. RECOVERY trial closes recruitment to colchicine treatment for patients hospitalised with COVID-19<br>Baricitinib in patients admitted to hospital with COVID-19 (RECOVERY): a randomised, controlled, open-label, platform trial and updated meta-analysis**,**RECOVERY Collaborative Group<br><br>Horby W. P., Emberson R. J. et al. Baricitinib in patients admitted to hospital with COVID-19 (RECOVERY): a randomised, controlled, open-label, platform trial and updated meta-analysis<br>Dimethyl fumarate in patients admitted to hospital with COVID-19 (RECOVERY): a randomised, controlled, open-label, platform trial, RECOVERY Collaborative Group
The REMAP-CAP (Randomized Embedded Multifactorial Adaptive Platform for Community-acquired Pneumonia) Study. Rationale and Design	
**Acronym**	REMAP-CAP
**Registry Number**	NCT02735707, 2015-002340-14, ISRCTN67000769
**Master protocol**	Curtis R. J., Kross K. E. et al. The Importance of Addressing Advance Care Planning and Decisions About Do-Not-Resuscitate Orders During Novel Coronavirus 2019 (COVID-19)<br><br>REMAP-CAP (REMAP-COVID) – Analysis of COVID-19 Immune Modulation Domain, May 30, 2021
**Results**	Angus D. C., Derde L. et al. Effect of Hydrocortisone on Mortality and Organ Support in Patients With Severe COVID-19The REMAP-CAP COVID-19 Corticosteroid Domain Randomized Clinical Trial<br>Arabi M. Y., Gordon C. A. et al. Lopinavir-ritonavir and hydroxychloroquine for critically ill patients with COVID-19: REMAP-CAP randomized controlled trial<br><br>https://www.remapcap.eu/2021/05/21/interleukin-6-receptor-antagonists-in-critically-ill-patients-with-covid-19/

© 2024 Griessbach A et al. *JAMA Network Open.*

	Iterleukin-6 Receptor Antagonists in Critically Ill Patients with Covid-19,The REMAP-CAP Investigators*<br>Estcourt J. L., Turgeon F. A. et al. Convalescent Plasma in Critically ill Patients with Covid-19<br>https://www.recover-europe.eu/press-release-antiplatelet-therapy-not-effective-in-treating-critically-ill-covid-19-patients/<br>Antiplatelet therapy not effective in treating critically ill COVID-19 patients, The REMAP-CAP Investigators
Accelerating clinical evaluation of repurposed combination therapies for COVID-19	
**Acronym**	SOLIDARITY
**Registry Number**	NCT04647669, SRCTN83971151
**Master protocol**	Not available
**Results**	Publication: Pan H., Peto R. et al. Repurposed antiviral drugs for COVID-19 –interim WHO SOLIDARITY trial results<br>Pre-print : Pan H., Peto R. et al. Repurposed Antiviral Drugs for Covid-19 — Interim WHO Solidarity Trial Results<br>Remdesivir and three other drugs for hospitalised patients with COVID-19: final results of the WHO Solidarity randomised trial and updated meta-analyses, Solidarity Trial Consortium
Repurposing Metformin as Therapy for Prostate Cancer within the STAMPEDE Trial Platform	
**Acronym**	STAMPEDE
**Registry Number**	NCT00268476, 2004-000193-31, ISRCTN78818544
**Master protocol**	Parker C. C., Sydes R. M. et al. Prostate radiotherapy for men with metastatic disease: a new comparison in the Systemic Therapy in Advancing or Metastatic Prostate Cancer: Evaluation of Drug Efficacy (STAMPEDE) trial<br>Sydes R. M., Parmar K. B. M. et al. Flexible trial design in practice - stopping arms for lack-of-benefit and adding research arms mid-trial in STAMPEDE: a multi-arm multi-stage randomized controlled trial<br>Attard G., Sydes R. M. et al. Combining Enzalutamide with Abiraterone, Prednisone, and Androgen Deprivation Therapy in the STAMPEDE Trial<br>Parmar B. K. M., Sydes M. et al. Speeding up the Evaluation of New Agents in Cancer<br>Gillessen et al, Repurposing Metformin as Therapy for Prostate Cancer within the STAMPEDE Trial Platform<br>Sydes R. M., Parmar K. B. M. et al. Issues in applying multi-arm multi-stage methodology to a clinical trial in prostate cancer: the MRC STAMPEDE trial
**Results**	James et al., Survival with Newly Diagnosed Metastatic Prostate Cancer in the “Docetaxel Era”: Data from 917 Patients in the Control Arm of the STAMPEDE Trial (MRC PR08, CRUK/06/019)<br>James D. N., Spears R. M. et al. Failure-Free Survival and Radiotherapy in Patients With Newly Diagnosed Nonmetastatic Prostate Cancer, Data From Patients in the Control Arm of the STAMPEDE Trial<br>James D. N., Addition of docetaxel, zoledronic acid, or both to first-line long-term hormone therapy in prostate cancer (STAMPEDE): survival results from an adaptive, multiarm, multistage, platform randomised controlled trial<br>Mason et al., Adding Celecoxib With or Without Zoledronic Acid for Hormone-Naïve Prostate Cancer: Long-Term Survival Results From an Adaptive, Multiarm, Multistage, Platform, Randomized Controlled Trial<br>James D. N., Celecoxib plus hormone therapy versus hormone therapy alone for hormone-sensitive prostate cancer: first results from the STAMPEDE multiarm, multistage, randomised controlled trialMason D. M., Clarke W. N. et al. Adding Celecoxib With or Without Zoledronic Acid for Hormone-Naïve Prostate Cancer: Long-Term Survival Results From an Adaptive, Multiarm, Multistage, Platform, Randomized Controlled Trial<br>James D. N., Abiraterone for Prostate Cancer Not Previously Treated with Hormone Therapy<br>Parker C. C., James D. N. et al. Radiotherapy to the primary tumour for newly diagnosed, metastatic prostate cancer (STAMPEDE): a randomised controlled phase 3 trial<br>Attard G., Murphy L. et al. Abiraterone acetate and prednisolone with or without enzalutamide for high-risk non-metastatic prostate cancer: a meta-analysis of primary results from two randomised controlled phase 3 trials of the STAMPEDE platform protocol<br>Website: https://Acronym.cancerresearchuk.org/about-cancer/find-a-clinical-trial/a-trial-looking-at-hormone-therapy-with-other-treatments-for-prostate-cancer-stampede-trial-results#undefined
STELLAR: A phase II, randomiSed study of CHOP-R in combination with acalabruTinib comparEd to CHOP-R in patients with newLy diagnosed Richter	
**Acronym**	STELLAR
**Registry Number**	NCT03899337, 2017-004401-40, ISRCTN52839057
**Master protocol**	Appleby N., Eyre A. T. et al. The STELLAR trial protocol: a prospective multicentre trial for Richter’s syndrome consisting of a randomised trial investigation CHOP-R with or without acalabrutinib for newly diagnosed RS and a single-arm platform study for evaluation of novel agents in relapsed disease
**Results**	Not available

© 2024 Griessbach A et al. *JAMA Network Open.*

mulTi-Arm Therapeutic Study in Pre-ICu Patients Admitted With Covid-19 - Experimental Drugs and Mechanisms (TACTIC-E)	
**Acronym**	TACTIC-E
**Registry Number**	NCT04393246, 2020-002229-27, CTRI/2021/05/033546
**Master protocol**	Lu N. I., Kulkarni S. et al. muLTi-Arm Therapeutic study in pre-ICu patients admitted with Covid-19-Experimental drugs and mechanisms (TACTIC-E): A structured summary of a study protocol for a randomized controlled trial
**Results**	Not available
Repurposed immunomodulatory drugs for Covid-19 in pre-ICu patients - mulTi-Arm Therapeutic study in pre-ICu patients admitted with Covid-19 - Repurposed Drugs (TACTIC-R): A structured summary of a study protocol for a randomised controlled trial	
**Acronym**	TACTIC-R
**Registry Number**	NCT04390464, 2020-001354-22, ISRCTN11188345
**Master protocol**	Kulkarni S., Fisk M. et al. Repurposed immunomodulatory drugs for Covid-19 in pre-ICu patients - mulTi-Arm Therapeutic study in pre-ICu patients admitted with Covid-19 – Repurposed Drugs (TACTIC-R): A structured summary of a study protocol for a randomised controlled trial
**Results**	Not available
TASTER- TArgeting STEm cell Resistance An umbrella adaptive randomised multi-arm screening phase II trial for patients with 2nd/3rd generation TKI resistant chronic myeloid leukaemia	
**Acronym**	TASTER
**Registry Number**	2018-001843-29, ISRCTN68270067
**Master protocol**	Not available
**Results**	Not available
Randomized, Embedded, Multifactorial, Adaptive Platform Trial for Optimizing Surgical Outcomes at UPMC	
**Acronym**	SPRY -UPMC REMAP
**Registry Number**	NCT03861767
**Master protocol**	Reitz M. K., Seymour W. C. et al. Strategies to Promote ResiliencY (SPRY): a randomised embedded multifactorial adaptative platform (REMAP) clinical trial protocol to study interventions to improve recovery after surgery in high-risk patients
**Results**	Not available
UPMC REMAP: Randomized, Embedded, Multifactorial Adaptive Platform Trial for Surgical Pathways Enhancing Recovery (SUPER) - Enhanced Recovery Protocols (ERP)	
**Acronym**	UPMC REMAP (EHR protocol)
**Registry Number**	NCT04606264
**Master protocol**	Not available
**Results**	Not available
Randomised Controlled Trials to Assess Approved SARS-CoV-2 Vaccines in Immunocompromised Patients: A Master Protocol for the Set-up of a Swiss Cohorts Based Trial Platform	
**Acronym**	COVERALL
**Registry Number**	NCT04805125
**Master protocol**	Speich B., Chammartin F. et al. A trial platform to assess approved SARS-CoV-2 vaccines in immunocompromised patients: first sub-protocol for a pilot trial comparing the mRNA vaccines Comirnaty® and COVID-19 mRNA Vaccine Moderna®
**Results**	Speich B., Chammartin F. et al. Antibody Response in Immunocompromised Patients After the Administration of Severe Acute Respiratory Syndrome Coronavirus 2 (SARS-CoV-2) Vaccine BNT162b2 or mRNA-1273: A Randomized Controlled Trial
CONNECTS Master Protocol for Clinical Trials Targeting Macro-, Micro-immuno-thrombosis, Vascular Hyperinflammation, and Hypercoagulability and Renin-angiotensin-aldosterone System (RAAS) in Hospitalized Patients With COVID-19 (ACTIV-4 Host Tissue)	
**Acronym**	ACTIV-4 Host Tissue
**Registry Number**	NCT04924660
**Master protocol**	Not available
**Results**	Not available
ACTIV-6: COVID-19 Outpatient Randomized Trial to Evaluate Efficacy of Repurposed Medications	
**Acronym**	ACTIV-6
**Registry Number**	NCT04885530
**Master protocol**	Not available
**Results**	Naggie S., Boulware R. D. et al. Effect of Ivermectin vs Placebo on Time to Sustained Recovery in Outpatients With Mild to Moderate COVID-19A Randomized Clinical Trial<br>Naggi S., Boulware R. D. et al. Effect of Ivermectin 600 μg/kg for 6 days vs Placebo on Time to Sustained Recovery in Outpatients with Mild to Moderate COVID-19: A Randomized Clinical Trial<br>Naggi S. Ivermectin for Treatment of Mild-to-Moderate COVID-19 in the Outpatient Setting: A Decentralized, Placebo-controlled, Randomized, Platform Clinical Trial<br>McCarthy et al, Fluvoxamine for Outpatient Treatment of COVID-19: A Decentralized, Placebo-controlled, Randomized, Platform Clinical Trial
MORPHEUS: A Study Evaluating the Efficacy and Safety of Multiple Immunotherapy-Based Treatment Combinations in Patients With Metastatic or Inoperable Locally Advanced Triple-Negative Breast Cancer	
**Acronym**	MORPHEUS-TNBC
**Registry Number**	NCT03424005, 2017-002038-21

© 2024 Griessbach A et al. *JAMA Network Open.*

**Master protocol**	Yardley D. A., Abu-Khalaf M. et al. Abstract OT2-06-04: MORPHEUS: A phase Ib/II trial platform evaluating the safety and efficacy of multiple cancer immunotherapy combinations in patients with hormone receptor–positive and triple-negative breast cancer<br>Manji G., Bahary N. et al. 259 Phase Ib/II open-label, randomized evaluation of atezolizumab (atezo) + selicrelumab (seli) + gemcitabine+nab-paclitaxel (gem+nabP) or bevacizumab (bev) vs control in MORPHEUS-PDAC, -TNBC and -CRC
**Results**	Manji G., Bahary N. et al. 259 Phase Ib/II open-label, randomized evaluation of atezolizumab (atezo) + selicrelumab (seli) + gemcitabine+nab-paclitaxel (gem+nabP) or bevacizumab (bev) vs control in MORPHEUS-PDAC, -TNBC and -CRC
MORPHEUS: A Study of Multiple Immunotherapy-Based Treatment Combinations in Hormone Receptor (HR)-Positive Human Epidermal Growth Factor Receptor 2 (HER2)-Negative Breast Cancer	
**Acronym**	MORPHEUS HR+BC
**Registry Number**	NCT03280563
**Master protocol**	Yardley D. A., Abu-Khalaf M. et al. Abstract OT2-06-04: MORPHEUS: A phase Ib/II trial platform evaluating the safety and efficacy of multiple cancer immunotherapy combinations in patients with hormone receptor–positive and triple-negative breast cancer<br>Hurvitz A. S., Boni V. et al. Abstract PD10-04: Phase Ib/II open-label, randomized trial of atezolizumab (atezo) with ipatasertib (ipat) and fulvestrant (fulv) vs control in MORPHEUS-HR+ breast cancer (M-HR+ BC) and atezo with ipat vs control in MORPHEUS triple negative breast cancer (M-TNBC)
**Results**	Sonnenblick A., Im S-A. et al. 267P Phase Ib/II open-label, randomized evaluation of second- or third-line (2L/3L) atezolizumab (atezo) + entinostat (entino) in MORPHEUS-HR+ breast cancer (M-HR+BC)
MORPHEUS: A Study of Multiple Immunotherapy-Based Treatment Combinations in Patients With Locally Advanced Unresectable or Metastatic Gastric or Gastroesophageal Junction Cancer (G/GEJ) or Esophageal Cancer	
**Acronym**	MORPHEUS-GC
**Registry Number**	NCT03281369, 2016-004529-17
**Master protocol**	Manji A. G., Bendell C. J. et al. MORPHEUS: A phase Ib/II multi-trial platform evaluating the efficacy and safety of cancer immunotherapy (CIT)-based combinations in patients (pts) with gastric or pancreatic cancer.<br>Oh D., Al-Batran S. et al. MORPHEUS: A phase Ib/II trial platform evaluating the safety and efficacy of multiple cancer immunotherapy (CIT) combinations in patients (pts) with gastric or pancreatic cancer.<br>Desai J., Kortmansky S. J. et al. MORPHEUS: A phase Ib/II study platform evaluating the safety and clinical efficacy of cancer immunotherapy (CIT)–based combinations in gastrointestinal (GI) cancers.<br>Lewis K. D., Fury M. G. et al. Phase II study of cemiplimab, a human monoclonal anti-PD-1, in patients with advanced basal cell carcinoma (BCC) who experienced progression of disease on, or were intolerant of prior hedgehog pathway inhibitor (HHI) therapy
**Results**	Ko H. A., Lee J. et al. Phase Ib/II open-label, randomized evaluation of 2L atezolizumab (atezo) + PEGPH20 versus control in MORPHEUS-pancreatic ductal adenocarcinoma (M-PDAC) and MORPHEUS-gastric cancer (M-GC).<br>Ko H. A., Lee J. et al. Phase Ib/II open-label, randomized evaluation of 2L atezolizumab (atezo) + PEGPH20 versus control in MORPHEUS-pancreatic ductal adenocarcinoma (M-PDAC) and MORPHEUS-gastric cancer (M-GC).<br>Cho C. B., Bahary N. et al. Abstract CT201: Phase Ib/II open-label, randomized evaluation of atezolizumab + cobimetinib vs control in MORPHEUS-NSCLC (non-small cell lung cancer), MORPHEUS-PDAC (pancreatic ductal adenocarcinoma) and MORPHEUS-GC (gastric cancer)<br>Oh D., Ajani A. J. et al. Phase Ib/II open-label, randomized evaluation of 2L atezolizumab (atezo) + BL-8040 versus control in MORPHEUS-pancreatic ductal adenocarcinoma (M-PDAC) and MORPHEUS-gastric cancer (M-GC).<br>Lee J., Ajani J. A. et al. 1382P Phase Ib/II open-label, randomised evaluation of second-line atezolizumab (atezo) + linagliptin (lina) vs ramucirumab (ram) + paclitaxel (pac) in MORPHEUS-gastric cancer
MORPHEUS: A Study of Multiple Immunotherapy-Based Treatment Combinations in Participants With Metastatic Pancreatic Ductal Adenocarcinoma	
**Acronym**	MORPHEUS-PDAC
**Registry Number**	NCT03193190, 2016-004126-42
**Master protocol**	Manji A. G., Bendell C. J. et al. MORPHEUS: A phase Ib/II multi-trial platform evaluating the efficacy and safety of cancer immunotherapy (CIT)-based combinations in patients (pts) with gastric or pancreatic cancer.<br>Oh D., Al-Batran S. et al. MORPHEUS: A phase Ib/II trial platform evaluating the safety and efficacy of multiple cancer immunotherapy (CIT) combinations in patients (pts) with gastric or pancreatic cancer.<br>Desai J., Kortmansky S. J. et al. MORPHEUS: A phase Ib/II study platform evaluating the safety and clinical efficacy of cancer immunotherapy (CIT)–based combinations in gastrointestinal (GI) cancers.

© 2024 Griessbach A et al. *JAMA Network Open.*

**Results**	Ko H. A., Lee J. et al. Phase Ib/II open-label, randomized evaluation of 2L atezolizumab (atezo) + PEGPH20 versus control in MORPHEUS-pancreatic ductal adenocarcinoma (M-PDAC) and MORPHEUS-gastric cancer (M-GC).<br>Manji G., Bahary N. et al. 259 Phase Ib/II open-label, randomized evaluation of atezolizumab (atezo) + selicrelumab (seli) + gemcitabine+nab-paclitaxel (gem+nabP) or bevacizumab (bev) vs control in MORPHEUS-PDAC, -TNBC and -CRC<br>Cho C. B., Bahary N. et al. Abstract CT201: Phase Ib/II open-label, randomized evaluation of atezolizumab + cobimetinib vs control in MORPHEUS-NSCLC (non-small cell lung cancer), MORPHEUS-PDAC (pancreatic ductal adenocarcinoma) and MORPHEUS-GC (gastric cancer)<br>Ko H. A., Lee J. et al. Phase Ib/II open-label, randomized evaluation of 2L atezolizumab (atezo) + PEGPH20 versus control in MORPHEUS-pancreatic ductal adenocarcinoma (M-PDAC) and MORPHEUS-gastric cancer (M-GC).<br>Oh D., Ajani A. J. et al. Phase Ib/II open-label, randomized evaluation of 2L atezolizumab (atezo) + BL-8040 versus control in MORPHEUS-pancreatic ductal adenocarcinoma (M-PDAC) and MORPHEUS-gastric cancer (M-GC).<br>Chung et al., SO-4 phase Ib/II, open-label, randomised evaluation of atezolizumab plus RO6874281 vs control in MORPHEUS–pancreatic ductal adenocarcinoma
MORPHEUS: A Study Of Multiple Immunotherapy-Based Treatment Combinations In Participants With Metastatic Non-Small Cell Lung Cancer	
**Acronym**	MORPHEUS-Lung
**Registry Number**	NCT03337698, 2017-001267-21
**Master protocol**	Johnson L. M., Solomon J. B. et al. MORPHEUS: A phase Ib/II multi-trial platform evaluating the safety and efficacy of cancer immunotherapy (CIT)-based combinations in patients (pts) with non-small cell lung cancer (NSCLC)<br>Lewis K.D., Fury M.G. et al. Phase II study of cemiplimab, a human monoclonal anti-PD-1, in patients with advanced basal cell carcinoma (BCC) who experienced progression of disease on, or were intolerant of prior hedgehog pathway inhibitor (HHI) therapy
**Results**	Cho C. B., Bahary N. et al. Abstract CT201: Phase Ib/II open-label, randomized evaluation of atezolizumab + cobimetinib vs control in MORPHEUS-NSCLC (non-small cell lung cancer), MORPHEUS-PDAC (pancreatic ductal adenocarcinoma) and MORPHEUS-GC (gastric cancer)
MORPHEUS: A Study Evaluating the Efficacy and Safety of Multiple Immunotherapy-Based Treatment Combinations in Patients With Metastatic Colorectal Cancer	
**Acronym**	MORPHEUS-CRC
**Registry Number**	NCT03555149, 2017-004566-99
**Master protocol**	Desai J., Kortmansky S. J. et al. MORPHEUS: A phase Ib/II study platform evaluating the safety and clinical efficacy of cancer immunotherapy (CIT)–based combinations in gastrointestinal (GI) cancers.
**Results**	Desai J., Fakih M. et al. Phase Ib/II open-label, randomized evaluation of efficacy and safety of atezolizumab plus isatuximab versus regorafenib in MORPHEUS-colorectal cancer.<br>Fakih M., Cleary M J. et al. Phase Ib/II open-label, randomized evaluation of atezolizumab (atezo) + Imprime PGG (Imprime) + bevacizumab (bev) vs regorafenib (rego) in MORPHEUS: Microsatellite-stable (MSS) metastatic colorectal cancer (mCRC).<br>Manji G., Bahary N. et al. 259 Phase Ib/II open-label, randomized evaluation of atezolizumab (atezo) + selicrelumab (seli) + gemcitabine+nab-paclitaxel (gem+nabP) or bevacizumab (bev) vs control in MORPHEUS-PDAC, -TNBC and -CRC
MORPHEUS: Study Evaluating the Efficacy and Safety of Multiple Immunotherapy-Based Treatments and Combinations in Patients With Urothelial Carcinoma	
**Acronym**	MORPHEUS-UC
**Registry Number**	NCT03869190
**Master protocol**	Drakaki A., Rezazadeh A. et al. Phase Ib/II umbrella trial to evaluate the safety and efficacy of multiple 2L cancer immunotherapy (CIT) combinations in advanced/metastatic urothelial carcinoma (mUC): MORPHEUS-mUC.
**Results**	Not available
Finding Treatments for COVID-19: A Phase 2 Multi-centre Adaptive Platform Trial to Assess Antiviral Pharmacodynamics in Early Symptomatic COVID-19 (PLATCOV)	
**Acronym**	PLATCOV
**Registry Number**	NCT05041907
**Master protocol**	Schilling W. HK., Jittamala P. et al. Pharmacometric assessment of the *in vivo* antiviral activity of ivermectin in early symptomatic COVID-19<br>the latest version is on GitHub https://github.com/jwatowatson/PLATCOV-SAP/tree/main/Analysis_Plan
**Results**	Schillling et al, Pharmacometric assessment of the in vivo antiviral activity of ivermectin in early symptomatic COVID-19<br>Jittamala et al, Clinical antiviral efficacy of remdesivir and casirivimab/imdevimab against the SARS-CoV-2 Delta and Omicron variants
Australasian COVID-19 Trial (ASCOT) ADAptive Platform Trial	
**Acronym**	ASCOT ADAPT
**Registry Number**	NCT04483960, 2020-005963-29, ACTRN12620000445976
**Master protocol**	Not available

© 2024 Griessbach A et al. *JAMA Network Open.*

**Results**	Denholm J. T., Venkatesh B. et al. ASCOT ADAPT study of COVID-19 therapeutics in hospitalised patients: an international multicentre adaptive platform trial<br><br>McQuilten Z. K., Venkatesh B. et al. Anticoagulation Strategies in Non–Critically Ill Patients with Covid-19
Study of the Efficacy and Safety of Various Anti-inflammatory Agents in Participants With Mild Cognitive Impairment or Mild Alzheimer's Disease	
**Acronym**	EXPLAIN-AD
**Registry Number**	NCT04795466, 2020-003966-38
**Master protocol**	Not available
**Results**	Not available
HElping Alleviate the Longer-term Consequences of COVID-19 (HEAL-COVID)	
**Acronym**	HEAL-COVID
**Registry Number**	NCT04801940, 2021-001187-25, ISRCTN15851697
**Master protocol**	On registry (ISRCTN) and on website https://www.heal-covid.net/for-staff/staff-page/
**Results**	Toshner et al., Apixaban following discharge in hospitalised adults with COVID-19: Preliminary results from a multicentre, open-label, randomised controlled platform clinical trial,
Early Treatment of Vulnerable Individuals With Non-Severe SARS-CoV-2 Infection	
**Acronym**	COVERAGE-A
**Registry Number**	NCT04920838
**Master protocol**	Not available
**Results**	Not available
Study of NIS793 and Other Novel Investigational Combinations With SOC Anti-cancer Therapy for the 2L Treatment of mCRC	
**Acronym**	daNIS-3
**Registry Number**	NCT04952753, 2021-000553-40
**Master protocol**	Segal N., Rivera F. P-23 Phase II study (daNIS-3) of the anti–TGF-β monoclonal antibody NIS793 and other new investigational drug combinations with standard-of-care therapy vs standard-of-care alone in patients with second-line metastatic colorectal cancer
**Results**	Not available
Staphylococcus Aureus Network Adaptive Platform Trial	
**Acronym**	SNAP
**Registry Number**	NCT05137119
**Master protocol**	Not available
**Results**	Not available
A clinical trial investigating novel treatments for COVID-19 in the community	
**Acronym**	PANORAMIC
**Registry Number**	2021-005748-31, ISRCTN30448031
**Master protocol**	Not available
**Results**	Not available
Solidarity trial of candidate vaccines against COVID-19	
**Acronym**	SOLIDARITY Vaccine Trial
**Registry Number**	ISRCTN15779782
**Master protocol**	Not available
**Results**	Not available
A study to evaluate diagnostic tests used at the point of care for improving antibiotic prescribing for patients with respiratory tract infections in primary care in Europe	
**Acronym**	PRUDENCE
**Registry Number**	ISRCTN13336322
**Master protocol**	ISRCTNregistry, Platform randomized controlled trial of point of care diagnostics for enhancing the quality of antibiotic prescribing for community acquired acute respiratory tract infection in ambulatory care in Europe (PRUDENCE)
**Results**	Not available
A Study of Combination Therapies With Pembrolizumab (MK-3475) in Participants With Advanced Esophageal Cancer (MK-3475-06A)	
**Acronym**	KEYNOTE U06
**Registry Number**	NCT05319730, NCT05342636, 2021-005443-76, 2021-005405-26
**Master protocol**	Not available
**Results**	Not available
A Study Validating the Use of Candin as a Challenge Agent in Healthy Participants	
**Acronym**	PLATFORM (Candin)
**Registry Number**	NCT04589026, NCT04611971, NCT04985955, 2020-002480-59, 2020-002481-14, 2020-006066-37
**Master protocol**	Not available
**Results**	Not available
Combination Therapy for the Treatment of Diffuse Midline Gliomas	
**Acronym**	---
**Registry Number**	NCT05009992
**Master protocol**	Not available
**Results**	Not available

Platform Trial to Compare Homologous Boost of Authorized COVID-19 Vaccines and Heterologous Boost With UB-612 Vaccine	
**Acronym**	---
**Registry Number**	NCT05293665, 2022-000088-38
**Master protocol**	Not available
**Results**	Not available
An open-label, multicentre, randomised, adaptive platform trial of the safety and efficacy of several therapies, including antiviral therapies, versus control in mild/moderate cases of COVID-19	
**Acronym**	ANTICOV
**Registry Number**	PACTR202006537901307 Immuno Substudy: PACTR202010781639956, CTRI/2022/01/039235
**Master protocol**	Master protocol: https://anticov.org/wp-content/uploads/2021/07/ANTICOV-IMMUNO-StudyProtocol.pdf<br><br>Pan African Clinical Trials Registry, An open-label, multicentre, randomised, adaptive platform trial of the safety and efficacy of several therapies, including antiviral therapies, versus control in mild/moderate cases of COVID-19<br>Moncunill G., Adriaensen W. et al. The impact of COVID-19 treatment on the type, strength and duration of antibody and cellular immune responses in SARS-CoV-2 patients in sub-Saharan Africa
**Results**	AntiCov, ANTICOV trial finds drug combination nitazoxanide + ciclesonide does not reduce risk of hospitalization in COVID-19 outpatients<br>https://anticov.org/press-releases/2022/anticov-trial-finds-drug-combination-nitazoxanide-ciclesonide-does-not-reduce-risk-of-hospitalization-in-covid-19-outpatients/
An adaptive multi-arm phase II trial of maintenance targeted therapy after chemotherapy in metastatic urothelial cancer	
**Acronym**	ATLANTIS
**Registry Number**	2015-003249-25, ISRCTN25859465
**Master protocol**	Jones R., Powles T. et al. An adaptive multi-arm phase II trial of maintenance targeted therapy after chemotherapy in advanced/metastatic urothelial cancer<br>Fulton B., Jones R. et al. ATLANTIS: a randomised multi-arm phase II biomarker-directed umbrella screening trial of maintenance targeted therapy after chemotherapy in patients with advanced or metastatic urothelial cancer
**Results**	Jones J. R., Hussain A. S. et al. A randomised, double blind, phase II clinical trial of maintenance cabozantinib following chemotherapy for metastatic urothelial carcinoma (mUC): Final analysis of the ATLANTIS cabozantinib comparison.
BEAT COVID-19: A Bayesian adaptive platform, randomised controlled Trial to evaluate the efficacy and safety of interventions for COVID-19.	
**Acronym**	BEAT-COVID
**Registry Number**	ACTRN12620000566932
**Master protocol**	Not available
**Results**	Not available
Randomised controlled trial of intrave0us nafamostat mesylate in COVID pneumonitis: Phase 1b/2a experimental study to investigate safety, Pharmacokinetics and Pharmacodynamics	
**Acronym**	DEFINE
**Registry Number**	NCT04473053, 2020-002230-32, ISRCTN14212905
**Master protocol**	Gaughan E., Quinn T. et al. Evaluation of new or repurposed treatments for COVID-19: protocol for the phase Ib/IIa DEFINE trial platform<br>Gaughan E., Quinn T. et al. DEFINE: A Phase IIa Randomised Controlled Trial to Evaluate Repurposed Treatments for COVID-19
**Results**	Quinn T. M., Gaughan E. E. et al. Randomised controlled trial of intravenous nafamostat mesylate in COVID pneumonitis: Phase 1b/2a experimental study to investigate safety, Pharmacokinetics and Pharmacodynamics<br>Gaughan E. E., Quinn T. M. et al. An Inhaled Galectin-3 Inhibitor in COVID-19 Pneumonitis: A Phase Ib/IIa Randomized Controlled Clinical Trial (DEFINE)
AMLM22/D2-The International Acute myeloid leukaemia (AML) Platform Consortium (IAPC) trial is a randomised, multi-arm study platform to compare the efficacy of experimental therapies versus standard of care in subjects with acute myeloid leukaemia in first complete remission.	
**Acronym**	IAPC
**Registry Number**	ACTRN12619000280101, ACTRN12621000246886, ACTRN12619000248167
**Master protocol**	Not available
**Results**	Not available
AMLM26- INTERCEPT (Investigating Novel Therapy to Target Early Relapse and Clonal Evolution as Pre-emptive Therapy in AML): A Multi-arm, Precision-based, Recursive, Platform Trial	
**Acronym**	INTERCEPT
**Registry Number**	ACTRN12621000439842, ACTRN12622000582752, ACTRN12621001265864
**Master protocol**	Not available
**Results**	Not available
A Platform Study of Combination Immunotherapy for the Neoadjuvant and Adjuvant Treatment of Patients With Surgically Resectable Adenocarcinoma of the Pancreas	
**Acronym**	---
**Registry Number**	NCT02451982

© 2024 Griessbach A et al. *JAMA Network Open.*

**Master protocol**	Not available
**Results**	Heumann R. T., Judkins C. et al. Neoadjuvant and adjuvant antitumor vaccination alone or combination with PD1 blockade and CD137 agonism in patients with resectable pancreatic adenocarcinoma.<br>Zheng L., Judkins C. et al. 812 Urelumab (anti-CD137 agonist) in combination with vaccine and nivolumab treatments is safe and associated with pathologic response as neoadjuvant and adjuvant therapy for resectable pancreatic cancer
A multi-arm multi-stage, multi-centre, phase III (MAMS) platform trial that aims to assess hypotheses against a common standard-of-care control arm for the management of people with glioblastoma.	
**Acronym**	MAGMA
**Registry Number**	ACTRN12620000048987
**Master protocol**	Kong Y. B., Sim H. et al. Multi-Arm GlioblastoMa Australasia (MAGMA): protocol for a multiarm randomised clinical trial for people affected by glioblastoma
**Results**	Not available
Motor Neuron Disease Systematic Multi-Arm Adaptive Randomised Trial (MND-SMART)	
**Acronym**	MND-SMART
**Registry Number**	NCT04302870, 2019-000099-41
**Master protocol**	Wong C., Dakin S. R. et al. Motor Neuron Disease Systematic Multi-Arm Adaptive Randomised Trial (MND-SMART): a multi-arm, multi-stage, adaptive, platform, phase III randomised, double-blind, placebo-controlled trial of repurposed drugs in motor neuron disease
**Results**	Not available
An Open-label, Phase II, Platform Trial Evaluating Safety and Efficacy of Multiple BI 754091 Anti-PD-1 Based Combination Registry Numberimens in PD-(L)1 na	
**Acronym**	---
**Registry Number**	NCT03697304, 2018-002344-81
**Master protocol**	Hussein A. M., Bendell C. J. et al. Platform trial of BI 754091, an anti-PD-1 antibody, in patients with previously treated advanced solid tumors: Combination with BI 836880, a VEGF/Ang2-blocking nanobody.
**Results**	Hussein A. M., Percent J. I. et al. Platform trial of ezabenlimab (BI 754091), an anti-PD-1 antibody, in patients (pts) with previously treated advanced solid tumors: Combination with BI 836880, a VEGF/Ang2-blocking nanobody.<br>Ulahannan V. S., Percent J. I. et al. Ezabenlimab (BI 754091), an anti-PD-1 antibody, in combination with BI 836880, a VEGF/Ang2-blocking nanobody, in patients (pts) with advanced colorectal cancer (CRC).<br>Johnson L. M., Patel R. M. et al. Safety of BI 754111, an anti-LAG-3 monoclonal antibody (mAb), in combination with BI 754091, an anti-PD-1 mAb, in patients with advanced solid tumors.
Launching a comparative effectiveness adaptive platform trial of monoclonal antibodies for COVID-19 in 21 days	
**Acronym**	UPMC OPTIMISE-C19
**Registry Number**	NCT04790786
**Master protocol**	Huang T. D., McCreary K. E. et al. The UPMC OPTIMISE-C19 (OPtimizing Treatment and Impact of Monoclonal antIbodieS through Evaluation for COVID-19) trial: a structured summary of a study protocol for an open-label, pragmatic, comparative effectiveness platform trial with response-adaptive randomization
**Results**	McCreary K. E., Bariola R. J. et al. The comparative effectiveness of COVID-19 monoclonal antibodies: A learning health system randomized clinical trial<br>Huang T. D., McCreary K. E. et al. Effectiveness of Casirivimab-Imdevimab and Sotrovimab During a SARS-CoV-2 Delta Variant SurgeA Cohort Study and Randomized Comparative Effectiveness Trial<br>McCreary K. E., Kip K. E. et al. Evaluation of Bebtelovimab for Treatment of Covid-19 During the SARS-CoV-2 Omicron Variant Era
Randomized phase 2 umbrella study of various neoadjuvant therapies for patients with muscle-invasive urothelial carci0ma of the bladder (MIBC) who are cisplatin-ineligible or refuse cisplatin therapy and undergoing radical cystectomy (Optimus)	
**Acronym**	OPTIMUS
**Registry Number**	NCT04586244
**Master protocol**	Necchi A., Zakharia Y. et al. Randomized phase 2 umbrella study of various neoadjuvant therapies for patients with muscle-invasive urothelial carcinoma of the bladder (MIBC) who are cisplatin-ineligible or refuse cisplatin therapy and undergoing radical cystectomy (Optimus).<br>Klaassen Z. ASCO GU 2022: OPTIMUS: Randomized Phase 2 Umbrella Study of Various Neoadjuvant Therapies for Patients With MIBC Who Are Cisplatin-Ineligible or Refuse Cisplatin Therapy and Undergoing Radical Cystectomy
**Results**	Not available
Platform Trial In COVID-19 Vaccine BOOsting (PICOBOO) - A single blinded, phase IV, adaptive randomised trial to evaluate the immunogenicity of COVID-19 booster vaccines in adults	
**Acronym**	PICOBOO
**Registry Number**	ACTRN12622000238774
**Master protocol**	Not available
**Results**	Not available

© 2024 Griessbach A et al. *JAMA Network Open.*

A Phase II, randomised, single-blind, platform trial to assess safety, reactogenicity and immunogenicity of COVID-19 vaccines in pregnant women in the United Kingdom	
**Acronym**	Preg-Cov
**Registry Number**	2021-003073-60, ISRCTN15279830
**Master protocol**	Not available
**Results**	Not available
TOGETHER 3 Trial: An Adaptive Randomized Platform Trial to Investigate the Efficacy of Novel Agents for Treatment of SARS-CoV-2 Infection Among High-Risk Outpatient Adults. COVID-19	
**Acronym**	TOGETHER
**Registry Number**	NCT04727424, PACTR202007700757139
**Master protocol**	Reis G., Silva M. S. A. E., et al. A multi-center, adaptive, randomized, platform trial to evaluate the effect of repurposed medicines in outpatients with early coronavirus disease 2019 (COVID-19) and high-risk for complications: the TOGETHER master trial protocol [version 2; peer review: 1 approved, 1 approved with reservations]
**Results**	Reis G., Silva M. S. A. E. et al. Effect of early treatment with fluvoxamine on risk of emergency care and hospitalisation among patients with COVID-19: the TOGETHER randomised, platform clinical trial<br>Reis G., Silva M. S. A. E. et al. Effect of Early Treatment with Ivermectin among Patients with Covid-19<br>Reis G., Silva M. S. A. E. et al. Effect of early treatment with metformin on risk of emergency care and hospitalization among patients with COVID-19: The TOGETHER randomized platform clinical trial
Phase 1b/2 umbrella study of investigational immune and targeted combination therapies for patients with advanced clear cell renal cell carci0ma (ccRCC)	
**Acronym**	U03
**Registry Number**	NCT04626479, NCT04626518, 2019-003609-84, 2019-003610
**Master protocol**	Motzer J. R., Albiges L. et al. Phase 1b/2 umbrella study of investigational immune and targeted combination therapies for patients with advanced clear cell renal cell carcinoma (ccRCC).<br>Plimack R. E., Hammers J. H. et al. A phase 1b/2 umbrella study of investigational immune and targeted combination therapies as first-line therapy for patients with advanced renal cell carcinoma (RCC).
**Results**	Not available
Finding the Optimal Registry Numberimen for Mycobacterium abscessus Treatment (FORMaT)	
**Acronym**	FORMaT
**Registry Number**	NCT04310930, ACTRN12618001831279
**Master protocol**	Not available
**Results**	Not available
Biochemical response (PSA0) and testosterone (T) recovery in Metacure, a multi-arm multi-modality (MM) therapy (tx) for very high risk localized (HRL) and low volume metastatic (LVM) prostatic ade0carci0ma	
**Acronym**	Metacure
**Registry Number**	NCT03436654
**Master protocol**	Teo Y. M., Taplin M. et al. Metacure: Multi-arm multimodality therapy for very high risk localized and low volume metastatic prostatic adenocarcinoma.
**Results**	Not available
REcovering From COVID-19 Lingering Symptoms Adaptive Integrative Medicine (RECLAIM) Trial	
**Acronym**	RECLAIM
**Registry Number**	NCT05513560
**Master protocol**	Not available
**Results**	Not available
SOLIDARITY TRIAL PLUS: An international randomized trial of additional treatments for COVID-19 in hospitalized patients who are all receiving the local standard of care	
**Acronym**	SOLIDARITY TRIAL PLUS
**Registry Number**	ISRCTN18066414
**Master protocol**	Solidarity Trial Plus World Health Organization, An international randomised trial of additional treatments for COVID-19 in hospitalised patients who are all receiving the local standard of care
**Results**	Not available
Better Evidence and Translation for Calciphylaxis	
**Acronym**	BEAT-Calci
**Registry Number**	NCT05019221
**Master protocol**	Not available
**Results**	Not available
PATRIC: Pragmatic Adaptive Trial for Respiratory Infections in Children	
**Acronym**	PATRIC
**Registry Number**	ACTRN12621000967886R, ACTRN126190003189
**Master protocol**	Not available
**Results**	Not available
BEAT CF: Pulmonary Exacerbations Treatment Platform - Backbone Antibiotics Domain: An evaluation of the comparative effectiveness of prescribing various standard of care, first-line Backbone Antibiotics in the management of pulmonary	

© 2024 Griessbach A et al. *JAMA Network Open.*

exacerbations requiring intensive therapy (PERIT) in children and adults with CF, with respect to their short-term improvement in lung function.	
**Acronym**	BEAT CF
**Registry Number**	ACTRN12622000950763, ACTRN12621000638831
**Master protocol**	Not available
**Results**	Not available
Biochemical response (PSA0) and testosterone (T) recovery in Metacure, a multi-arm multi-modality (MM) therapy (tx) for very high risk localized (HRL) and low volume metastatic (LVM) prostatic ade0carci0ma	
**Acronym**	Metacure
**Registry Number**	NCT03436654
**Master protocol**	Teo M.Y., Taplin M. et al. Metacure: Multi-arm multimodality therapy for very high risk localized and low volume metastatic prostatic adenocarcinoma.
**Results**	Not available

© 2024 Griessbach A et al. *JAMA Network Open.*
