# Supplementary material for: Characteristics, Progression, and Output of Randomized Platform Trials: A Systematic Review
Source: JAMA Netw Open. 2024 Mar 20;7(3):e243109. doi: 10.1001/jamanetworkopen.2024.3109 (PMC10955344; doi:10.1001/jamanetworkopen.2024.3109)
Supplement: Supplement 2. — Data Sharing Statement [file jamanetwopen-e243109-s002.pdf]

Data Sharing Statement

Griessbach. Characteristics, Progression, and Output of Randomized Platform Trials. *JAMA Netw Open*. Published March 20, 2024. doi:10.1001/jamanetworkopen.2024.3109

Data
Data available: No

Additional Information
Explanation for why data not available: Data can be requested upon request
